# Molecularly tagged genes and quantitative trait loci in cucumber with recommendations for QTL nomenclature

**DOI:** 10.1038/s41438-019-0226-3

**Published:** 2020-01-01

**Authors:** Yuhui Wang, Kailiang Bo, Xingfang Gu, Junsong Pan, Yuhong Li, Jinfeng Chen, Changlong Wen, Zhonghai Ren, Huazhong Ren, Xuehao Chen, Rebecca Grumet, Yiqun Weng

**Affiliations:** 1 Department of Horticulture, University of Wisconsin, Madison, WI 53706 USA; 2 Institute of Vegetables and Flowers, Chinese Academy of Agricultural Sciences, Beijing, 100081 China; 3 Department of Plant Sciences, Shanghai Jiaotong University, Shanghai, 200240 China; 4 Horticulture College, Northwest A&F University, Yangling, 712100 China; 5 Horticulture College, Nanjing Agricultural University, Nanjing, 210095 China; 6 Beijing Vegetable Research Center, Beijing Academy of Agricultural and Forestry Sciences, Beijing, 100097 China; 7 College of Horticulture Science and Engineering, Shandong Agricultural University, Tai’an, 271018 China; 8 College of Horticulture, China Agricultural University, Beijing, 100193 China; 9 College of Horticulture and Plant Protection, Yangzhou University, Yangzhou, 225009 China; 10 Department of Horticulture, Michigan State University, East Lansing, MI 48824 USA; 11 USDA-ARS Vegetable Crops Research Unit, 1575 Linden Dr., Madison, WI 53706 USA

**Keywords:** Plant breeding, Genetic markers

## Abstract

Cucumber, *Cucumis sativus* L. (2*n* = 2*x* = 14), is an important vegetable crop worldwide. It was the first specialty crop with a publicly available draft genome. Its relatively small, diploid genome, short life cycle, and self-compatible mating system offers advantages for genetic studies. In recent years, significant progress has been made in molecular mapping, and identification of genes and QTL responsible for key phenotypic traits, but a systematic review of the work is lacking. Here, we conducted an extensive literature review on mutants, genes and QTL that have been molecularly mapped or characterized in cucumber. We documented 81 simply inherited trait genes or major-effect QTL that have been cloned or fine mapped. For each gene, detailed information was compiled including chromosome locations, allelic variants and associated polymorphisms, predicted functions, and diagnostic markers that could be used for marker-assisted selection in cucumber breeding. We also documented 322 QTL for 42 quantitative traits, including 109 for disease resistances against seven pathogens. By alignment of these QTL on the latest version of cucumber draft genomes, consensus QTL across multiple studies were inferred, which provided insights into heritable correlations among different traits. Through collaborative efforts among public and private cucumber researchers, we identified 130 quantitative traits and developed a set of recommendations for QTL nomenclature in cucumber. This is the first attempt to systematically summarize, analyze and inventory cucumber mutants, cloned or mapped genes and QTL, which should be a useful resource for the cucurbit research community.

## Introduction

Cucumber, *Cucumis sativus* L., is among the most widely cultivated and consumed vegetable crops throughout the world. In 2017, cucumber was grown on 919,146 hectares with a total production of 83,753,861 tons worldwide, and China is the largest producer with 77.4%, and 54.4% total production and acreage of the world, respectively (www.fao.org/faostat/en/). Cucumber was the first among major horticulture crops with a publicly available draft genome. The small, diploid genome (~400 Mbp), annual growth habit, self-compatible mating system, and relatively short life cycle (~3 months from seed to seed) offer significant advantages for genetic studies. The development of high-quality draft genomes and high-density genetic maps, coupled with utilization of high-throughput genotyping methods have greatly accelerated genetic mapping and gene/QTL cloning in cucumber. The 2016 Cucumber Gene Catalog documented 199 simply inherited genes or major-effect QTL^1^. In recent years, many genes listed in the catalog as well as new ones have been molecularly characterized or fine mapped. Hundreds of QTL for horticulturally important traits have been identified. While a wealth of data has been accumulated, a systematic review and inventory of the mutants, molecularly characterized or tagged genes, and QTL for cucumber is lacking. In addition, the QTL names used in various studies are inconsistent and confusing. It is imperative to develop a community standard for assignment of QTL names. Therefore, the objectives of this article are to: (1) review cloned and fine mapped genes or major-effect QTL. (2) Develop recommendations for QTL nomenclature for future QTL mapping studies. (3) Inventory published QTL in cucumber.

## Genes conferring simply inherited traits

As of July 2019, candidate genes have been identified for 51 simply inherited traits in cucumber (Table 1). Genes for additional 30 traits have been fine mapped with the target loci delimited to <2.0 Mbp (Table 2). It should be pointed out that, we used “cloned gene” in this review not in its strict term because for many mutants, identification of the candidate genes was based on genetic evidence, and their functions have not been validated or verified. Also, some major-effect QTL were counted as simply inherited genes, which often contribute to >20% observed phenotypic variance in QTL analysis. Details of the 81 genes are presented in supplementary File 1 (Table S1) including polymorphisms between the parents in the candidate gene, diagnostic markers, and primer sequences. Allelic variants for six genes (*cul*, *gl1*, *gl3*, *m*, *pm*, and *rl*) are listed separately in Table S1 (hence the total number is 88). The two variants of the *CsGL3* gene exhibit different phenotypes, which are listed as two genes. Three genes have names that are duplicated with previously reported ones including *glabrous2* (*gl2*)^2^, *ts* (*tender spine*)^3^, and *sf-1*(ref. ^4^), which were re-assigned *gl4*, *tsp*, and *sf-2*, respectively. The *CsSEP* gene was the candidate for a mutant with very long sepals^5^, which was assigned *els-1* (*extra-long sepal-1*) in this work.

**Table 1:** Details of identified genes for simply inherited traits in cucumber (as of July 2019).

#	Category	Sub-category	Gene and mutants^a^	Candidate gene (Gy14 V2.0)	Gy14 V2.0 Location	Variants/pleiotropy	Predicted functions
1	Abiotic stress tolerance	Waterlogging	*qARN6.1 (Adventitious roots number)*	*CsARN6.1 (CsGy6G030800.1)*	Chr6: 28825007		AAA ATPase domain-containing protein
2	Disease resistance	Bacterial resistance	*psl (Resistance to P. syringae pv. lachrymans)*	*CsSGR (CsGy5G003280.1)*	Chr5: 2149251		Staygreen (Mg dechelatase)
3	Disease resistance	Fungal resistance	*cla (Resistance to Colletotrichum lagenarium)*	*CsSGR (CsGy5G003280.1)*	Chr5: 2149251		Staygreen (Mg dechelatase)
4	Disease resistance	Fungal resistance	*pm5.1 (Resistance to Podosphaera fusca)*	*CsMLO1 (CsGy5G026660.1)*	Chr5: 30524541	Three haplotypes	Cell membrane protein of mildew locus O (MLO)
5	Disease resistance	Fungal resistance	*cca-3 (Resistance to Corynespora cassiicola)*	*cca-3 (CsGy6G019440.1)*	Chr6: 19877323		CC-NB-ARC type resistance homolog
6	Disease resistance	Oomycete resistance	*dm1 (Resistance to Pseudoperonospora cubensis)*	*CsSGR (CsGy5G003280.1)*	Chr5: 2149251		Staygreen (Mg dechelatase)
7	Disease resistance	Virus resistance	*zym (Resistance to Zucchini Yellow Mosaic Virus)*	*CsVPS4 (CsGy6G012710.1)*	Chr6: 10962805	*zym*^*A192-18*^*, zym*^*Dina*^*, zym*^*TMG1*^	Vacuolar protein sorting-associated protein 4 (VPS4)-like
8	Vegetative organs	Hypocotyl	*sh1 (Short hypocotyl1)*	*CsSH1 (CsGy3G012350.1)*	Chr3: 9318259		Human SMARCA3 chromatin remodeler
9	Vegetative organs	Leaf	*vyl (Virescent yellow leaf)*	*CsVYL (CsGy4G021760.1)*	Chr4: 28387102		DnaJ-like zinc finger protein
10	Vegetative organs	Leaf	*v-1 (Virescent leaf 1)*	*CsCNGCs (CsGy6G011700.1)*	Chr6: 10205338		Cyclic-nucleotide-gated ion channel protein
11	Vegetative organs	Leaf	*yp (Yellow plant; golden leaf)*	*CsChlI (CsGy6G034680.1)*	Chr6: 30831621		CHLI subunit of Mg-chelatase
12	Vegetative organs	Leaf	*rl-1 (Round leaf1)*	*CsPID (CsGy1G024130.1)*	Chr1: 22923411	*rl, rl-2*	Serine/threonine kinase
13	Vegetative organs	Leaf	*cul-1 (Curly leaf1)*	*CsPHB (CsGy6G036200.1)*	Chr6: 31806239	*cul-2*	HD-ZIP III transcription factor
14	Vegetative organs	Leaf	*ll (Littleleaf)*	*CsSAP (CsGy6G009260.1)*	Chr6: 7716895		WD40 repeat domain-containing protein
15	Vegetative organs	Leaf	*scl-1 (Small and cordate leaf)*	*CsSCL1 (CsGy7G005090.1)*	Chr7: 3797553		Nucleoside bisphosphate phosphatase
16	Vegetative organs	Leaf	*Psm (Paternal sorting of mitochondria)*	*CsPPR336 (CsGy3G036250.1)*	Chr3: 34459723		pentatricopeptide repeat (PPR) protein
17	Vegetative organs	Tendril	*ten (Tendril-less)*	*CsTEN (CsGy5G029420.1)*	Chr5: 32763879		TCP transcription factor
18	Vegetative organs	Architecture	*cp (compact)*	*CsCullin1 (CsGy6G014690.1)*	Chr6: 12880366		Cullin-1 protein
19	Vegetative organs	Architecture	*si (short internode)*	*CsVFB1 (CsGy4G022710.1)*	Chr4: 29079919		F-box protein, VIER F-BOX PROTEIN subfamily
20	Vegetative organs	Architecture	*scp-1 (Super compact1)*	*CsCYP85A1 (CsGy5G028960.1)*	Chr5: 32438112		BR-C6-oxidase
21	Vegetative organs	Architecture	*scp-2 (Super compact2)*	*CsDET2 (CsGy3G029480.1)*	Chr3: 29959460		steroid 5-alpha-reductase
22	Vegetative organs	Trichome	*gl1 (Glabrous1)*	*CsGL1 (CsGy3G031820.1)*	Chr3: 31245240	*mict (Micro-trichome)*	HD-ZIP I protein
23	Vegetative organs	Trichome	*gl3 (Glabrous3)*	*CsGL3 (CsGy6G033240.1)*	Chr6: 30057625	*tril (Trichome-less)*	HD-ZIP IV protein
24	Flower	Flowering time	*qEf1.1 (Early flowering time) (move to QTL)*	*CsFT (CsGy1G030960.1)*	Chr1: 29537849		Arabidopsis FLOWERING LOCUS T (FT) homolog
25	Flower	Male sterility	*ms-3 (Male sterility)*	*ms-3 (CsGy3G001080.1)*	Chr3: 804179		Homeodomain (PHD) finger protein
26	Flower	Flower structure	*els (Extra long sepal)*	*CsSEP2 (CsGy4G009560.1)*	Chr4:8004182		SEPALLATA2 (SEP2)
27	Flower	Sex expression	*F (Femaleness)*	*CsACS1G (CsGy6G028780.1)*	Chr6: 27588957		1-aminocyclopropane-1-carboxylic acid synthase (ACS)
28	Flower	Sex expression	*A (Androecious)*	*CsACS11 (CsGy2G018140.1)*	Chr2: 27954918		1-aminocyclopropane-1-carboxylic acid synthase (ACS)
29	Flower	Sex expression	*a-1 (Androecious-1)*	*CsACO2 (CsGy6G032740.1)*	Chr6: 29787615		1-aminocyclopropane-1-carboxylate (ACC) oxidase (ACO)
30	Flower	Sex expression	*m (Andromonoecious)*	*CsACS2 (CsGy1G027100.1)*	Chr1: 25520318	*m-1*	1-aminocyclopropane-1-carboxylic acid synthase (ACS)
31	Fruit	Size and shape	*cn (Carpel number)*	*CsCLV3 (CsGy1G014910.1)*	Chr1: 10816556		CLAVATA3
32	Fruit	Size and shape	*mf (Mango fruit)*	*CsWOX1 (CsGy1G007020.1)*	Chr1: 4488841		WOX1 (WUSCHEL-related homeobox1)
33	Fruit	Size and shape	*FS1.2 (Fruit size1.2)*	*CsSUN2 (CsGy1G026840.1)*	Chr1: 25331715		Tomato SUN homolog
34	Fruit	Size and shape	*FS2.1 (Fruit size2.1)*	*CsTRM4 (CsGy2G011350.1)*	Chr2: 11224424		AtTRM5/SlTRM5 (TON1 RECRUIT MOTIF) homolog
35	Fruit	Size and shape	*ful (Fruitful)*	*CsFUL1 (CsGy1G006040.1)*	Chr1:3947246		MADS-box gene
36	Fruit	Size and shape	*sf2 (Short fruit 2)*	*CsSF2 (CsGy2G010390.1)*	Chr2: 10113710		putative RING-type E3 ligase
37	Fruit	Peduncle direction	*up (upward-pedicel)*	*CsUP (CsGy1G024010.1)*	Chr1: 22736083		Auxilin-like protein with DnaJ-domain
38	Fruit	Epidermal feature	*lgp (Light green peel)*	*CsARC5 (CsGy7G004650.1)*	Chr7: 3474053		Accumulation and Replication of Chloroplasts 5(ARC5)
39	Fruit	Epidermal feature	*lgf (Light green fruit)*	*CsYcf54 (CsGy6G010900.1)*	Chr6: 9382904		Ycf54-like protein
40	Fruit	Epidermal feature	*w (White skin color)*	*CsAPRR2 (CsGy3G044470.1)*	Chr3: 41175650		two-component response regulator-like APRR2
41	Fruit	Epidermal feature	*B (Black spine)*	*CsMYB60 (CsGy4G001040.1)*	Chr4: 635650	Pleiotropic to *R*	R2R3-MYB transcription factor
42	Fruit	Epidermal feature	*fs1 (few spines)*	*CsGL3 (CsGy6G033240)*	Chr6: 30057625	*fsd6.2*	HD-ZIP IV protein
43	Fruit	Epidermal feature	*ns (Numerous spines*)	*CsLAX3 (CsGy2G013240.1)*	Chr2: 13010523		Auxin transporter-like protein 3
44	Fruit	Epidermal feature	*tsp (tender spines*)	*CsTspn (CsGy1G010080.1)*	Chr1: 6251039	*gl4*	C-type lectin receptor-like kinase
45	Fruit	Epidermal feature	*Ts1 (Tubercle size)*	*CsTbs1 (CsGy5G017890.1)*	Chr5: 24119025		Arabidopsis thaliana oleosin homolog
46	Fruit	Epidermal feature	*Tu (Warty)*	*CsTu (CsGy5G019590.1)*	Chr5: 25945387		C2H2 zinc finger domain-containing transcription factor
47	Fruit	Flesh color	*ore (Orange flesh)*	*CsBCH1 (CsGy3G017310.1)*	Chr3: 13300278		β-carotene hydroxylase
48	MISC	Bitterness	*bi (bitterfree)*	*CsBi (CsGy6G007190.1)*	Chr6: 6147641		Cucurbitadienol synthase
49	MISC	Bitterness	*bl (bitter leaf)*	*CsBl (CsGy5G003320.1)*	Chr5: 2175005		Basic helix-loop-helix (bHLH) transcription factor
50	MISC	Bitterness	*bt (bitter fruit)*	*CsBt (CsGy5G003340.1)*	Chr5: 2198903		Basic helix-loop-helix (bHLH) transcription factor
51	MISC	Fragrance	*fgr (Fragrance)*	*CsBADH (CsGy1G001790.1)*	Chr1: 1165392		Betainealdehyde dehydrogenase 2

^a^Complete references are provided in Supplementary File 1 (Table S1)

**Table 2:** List of fine mapped genes or major-effect QTL in cucumber (as of July 2019).

#	Category	Sub-category	Gene and mutants^a^	Gy14 V2.0 Location	Physical Interval^b^
1	Vegetative organ	Leaf	*vl (variegated leaf)*	Chr6:21297426	n/a
2	Vegetative organ	Leaf	*ll-2 (littleleaf -2)*	Chr7:1705258	1.24 Mb
3	Vegetative organ	Tendril	*td-1 (tendrilles-1)*	Chr6:32202841	190 kb
4	Vegetative organ	Trichome	*gl2 (Glabrous2)*	Chr2:20772692	0.6 cM
5	Vegetative organ	Trichome	*gl4 (gl2, glabrous2)*	Chr1:6247822	720 kb
6	Vegetative organ	Architecture	*cp-1 (compact1)*	Chr4:29878253	178 kb
7	Vegetative organ	Architecture	*dw (dwarf)*	Chr3:38398789	n/a
8	Fruit	Epidermal feature	*ygp (Yellow green peel)*	Chr2: 27932225	n/a
9	Fruit	Epidermal feature	*u (uniform immature fruit color)*	Chr5:25663570	313.2 kb
10	Fruit	Epidermal feature	*D (Dull fruit skin)*	Chr5:26438292	244.9 kb
11	Fruit	Epidermal feature	*H (Heavy netting)*	Chr5:25709527	1.2 Mb
12	Fruit	Epidermal feature	*Pe (Palisade epidermis)*	Chr5:25915175	227.5 kb
13	Fruit	Epidermal feature	*Fr (Fruit ribbing)*	Chr5:26431293	2.4 cM
14	Fruit	Epidermal feature	*Te (Tender fruit skin)*	Chr5:26000000^c^	n/a
15	Fruit	Epidermal feature	*ss (small spine)*	Chr5:25972294	189 kb
16	Fruit	Flesh	*yf (yellow flesh)*	Chr7:19537576	149 kb
17	Fruit	Flesh	*fth2.1 (Fruit flesh thickness2.1)*	Chr2: 4434893	190 kb
18	Fruit	Size and shape	*sf-1 (short fruit-1)*	Chr6:11696118	174.3 kb
19	Disease resistance	Fungal resistance	*Foc (Resistance F. oxysporum f. sp. Cucumerinum)*	Chr2:3276171	740 kb
20	Disease resistance	Fungal resistance	*pm1.1 (Resistance Podosphaera fusca)*	Chr1:6841559	41.1 kb
21	Disease resistance	Fungal resistance	*pm-s (Resistance Podosphaera fusca)*	Chr5:30406396	135.7 kb
22	Disease resistance	Fungal resistance	*pm5.3 (Resistance Podosphaera fusca)*	Chr5:30434472	468.0 kb
23	Disease resistance	Fungal resistance	*cca-1 (Resistance to Corynespora cassiicola)*	Chr6:17894751	2.9 cM
24	Disease resistance	Fungal resistance	*cca-2 (Resistance to Corynespora cassiicola)*	Chr6:9468049	1.25 Mb
25	Disease resistance	Fungal resistance	*ccu (Resistance to Cladosporium cucumerinum)*	Chr2:3276171	180 kb
26	Disease resistance	Oomycete resistance	*dm4.1 (Pseudoperonospora cubensise)*	Chr4:22679946	322 kb
27	Disease resistance	Oomycete resistance	*dm5.2 (Pseudoperonospora cubensis)*	Chr5:23380844	628 kb
28	Disease resistance	Virus resistance	*cmv6.1* (*Resistance to cucumber mosaic virus)*	Chr6:7688887	1.62 Mb
29	Disease resistance	Virus resistance	*PRSV* (*Resistance to Papaya ringspot virus)*	Chr6:9726336	1.8 cM
30	Disease resistance	Virus resistance	*wmv* (*Resistance to watermelon mosaic virus)*	Chr6:22530869	134.7 kb

^a^Complete reference is provided in Supplementary File 1 (Table S1)

^b^Estimated by flanking markers; n/a = not available or not applicable

^c^Estimated from the *Tender fruit* (*Te*) location

**Fig. 1:**
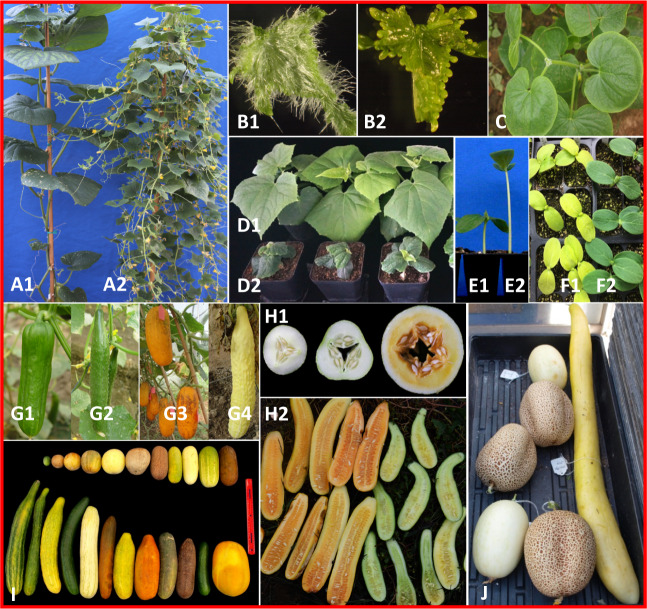
Phenotypes of representative mutants in cucumber. **a**–**f** shows mutant and wild-type phenotypes for *littleleaf* (*ll*, A2), *glabrous3* (*gl3*, B2), *roundleaf* (*rl*, **c**), *super compact-1* (*scp-*1, D2), *short hypocotyl1* (*sh1*, E1), *yellow plant* (*yp*, F1), respectively. **g** thru **j** show phenotypic variation in spine size and density (**g**), fruit flesh color (white, orange, yellow, and green), cavity size (**h**), fruit size, shape, and fruit epidermal features (**i**, **j**) in natural populations.

**Fig. 2:**
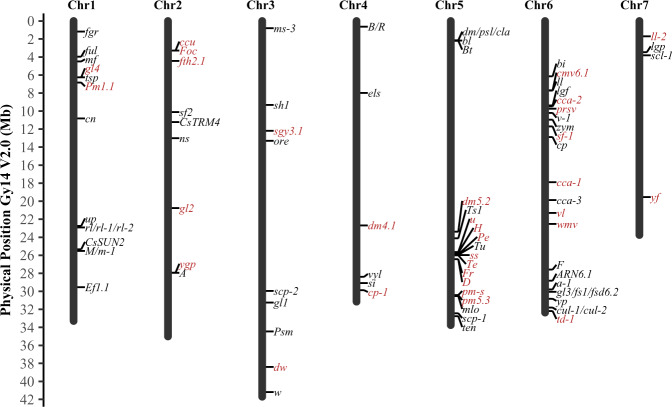
Chromosomal locations of 81 cloned (black) or fine mapped (red) genes in cucumber. Ruler to the left indicates locations (in Mbp) in the Gy14 V2.0 draft genome assembly (drawn to scale).

Among the 81 cloned or fine mapped genes or major-effect QTL, 14 are EMS-induced mutations, and the rest are spontaneous mutations identified from natural populations. Of the 51 cloned candidate genes, 42 mutants are due to SNPs; other polymorphisms include small or large deletions, and retrotransposon insertions. In most cases, the SNPs or insertions result in frame shift or amino acid substitutions, or alternate splicing (supplementary File 1). For convenience, the 81 genes/QTL were classified into six categories: Vegetative organ (23), Flower (7), Fruit (28), Disease resistance (18), Abiotic stress tolerance (1), and Miscellaneous (MISC) (4). Phenotypes of some representative plant architecture, leaf or fruit mutants are shown in Fig. 1. Distribution of the 81 genes or QTL across 7 cucumber chromosomes are illustrated in Fig. 2.

## Establishment of controlled vocabularies to describe quantitative traits and recommendations for QTL nomenclature in cucumber

Most horticulturally important traits in cucumber are controlled by QTL. With the exponential increase of QTL mapping studies in cucumber, one complicating issue is the naming of quantitative traits and corresponding QTL, which is currently very confusing. It is common that the same name was used for different traits or different names were used for the same trait. Thus, we reviewed the literature and phenotyping manuals from both public institutions and private seed companies. We also consulted colleagues in the cucumber research community and proposed the following rules for use of abbreviations to name quantitative traits in cucumber.

1. For disease/insect resistances: use common names except for *Fusarium* wilt and *Fusarium* crown rot, for which FOC and FCROS have been widely used, respectively.
2. For a trait name with one word, use first three letters.
3. For a trait name with two words, use the initial from each word. In a few cases, three letters (one from the initial of one word and two from another word) are used to avoid duplication with other traits, or for better understanding of its meaning.
4. For traits with more than two words, use the initial from each word.

Based on inputs from the community, 130 quantitative traits were identified. Their full names and recommended QTL names (abbreviations) are listed in Table 3. Considering the common practices taken by the cucurbit research community, we also recommend the following rules in assigning QTL names:

1. QTL name format: ***Trait name.chr#.QTL order on chromosome***.
2. When multiple QTL on the same chromosome (linkage group) are reported for the same trait, the numbering order follows the order of discovery in the literature.
3. The use of capital or lower case letters depends on the inheritance of the trait (dominant, co-dominant, or recessive).

**Table 3:** Proposed nomenclature for quantitative traits in QTL mapping studies in cucumber.

#	Category	Sub-category	Traits	Abbreviations
1	Abiotic stress tolerance	Chilling tolerance	Chilling Tolerance	CT
2	Abiotic stress tolerance	Low temperature germination	Low Temperature Germination	LTG
3	Abiotic stress tolerance	Drought tolerance	Water Deficit Tolerance	WDT
4	Abiotic stress tolerance	Heat tolerance	Heat Tolerance	HT
5	Abiotic stress tolerance	Waterlogging tolerance	Adventitious Root Number	ARN
6	Abiotic stress tolerance	Waterlogging tolerance	Waterlogging Tolerance	WLT
7	Abiotic stress tolerance	Sulfur tolerance	Sulfur Tolerance	ST
8	Disease resistance	Disease development	Chlorosis	CHL
9	Disease resistance	Disease development	Necrosis	NEC
10	Disease resistance	Disease development	Sporulation	SPR
11	Disease resistance	Bacterial resistance	Resistance to Angular Leaf Spot (*P. syringae pv. Lachryman*)	ALS
12	Disease resistance	Bacterial resistance	Resistance to Bacterial Wilt (*Erwinia tracheiphila*)	BW
13	Disease resistance	Fungal resistance	Resistance to Anthracnose (*Colletotrichum lagenarium*)	AN
14	Disease resistance	Fungal resistance	Resistance to Scab (*Cladosporium cucumerinum*)	SC
15	Disease resistance	Fungal resistance	Resistance to *Fusarium oxysporum* f. sp. c*ucumerinum* (Fusarium Wilt)	FOC
16	Disease resistance	Fungal resistance	Resistance to *F. oxysporum f. sp. radicis-cucumerinum*(Fusarium crown rot)	FCRO
17	Disease resistance	Fungal resistance	Resistance to Gray Mold (*Botrytis cinerea*)	GM
18	Disease resistance	Fungal resistance	Resistance to Gummy Stem Blight (*Didymella bryoniae*)	GSB
19	Disease resistance	Fungal resistance	Resistance to Phytophthora Fruit Rot (*Phytophthora capsici)*	PFR
20	Disease resistance	Fungal resistance	Resistance to Powdery Mildew (*Podosphaera fusca*)	PM
21	Disease resistance	Fungal resistance	Resistance to Target Leaf Spot (*Corynespora cassiicola*)	TLS
22	Disease resistance	Nematode resistance	Resistance to Java Rootknot Nematode (*Meloidogyne javanica*)	JRN
23	Disease resistance	Nematode resistance	Resistance to Southern Rootknot Nematode (*Meloidogyne incognita*)	SRN
24	Disease resistance	Oomycete resistance	Resistance to Downy Mildew (*Pseudoperonospora cubensis*)	DM
25	Disease resistance	Virus resistance	Resistance to Cucumber Green Mottle Mosaic Virus	CGMMV
26	Disease resistance	Virus resistance	Resistance to Cucumber Mosaic Virus	CMV
27	Disease resistance	Virus resistance	Resistance to Cucumber Vein Yellowing Virus	CVYV
28	Disease resistance	Virus resistance	Resistance to Cucurbit Yellow Stunting Disorder Virus	CYSDV
29	Disease resistance	Virus resistance	Resistance to Melon Yellow Spot Virus	MYSV
30	Disease resistance	Virus resistance	Resistance to Papaya Ringspot Virus	PRSV
31	Disease resistance	Virus resistance	Resistance to Tomato Leaf Curl New Delhi Virus	ToLCNDV
32	Disease resistance	Virus resistance	Resistance to Watermelon Mosaic Virus	WMV
33	Disease resistance	Virus resistance	Resistance to Zucchini Yellow Mosaic Virus	ZYMV
34	Insect resistance	Aphid	Resistance to melon/cotton aphid (*Aphis gossypii*)	MA
35	Insect resistance	Cucumber beetle	Resistance to Banded Cucumber Beetle (*Diabrotica balteata*)	BCB
36	Insect resistance	Cucumber beetle	Resistance to Spotted Cucumber Beetle (*Diabrotica undecimpunctata*)	SCB
37	Insect resistance	Cucumber beetle	Resistance to Striped Cucumber Beetle (*Acalymma vittatum*)	STB
38	Insect resistance	Leaf folder	Resistance to Leaf Folder (*Diaphania indica*)	LF
39	Insect resistance	Leaf miner	Resistance to Leaf Miner (Liriomyza huidobrensis)	LM
40	Insect resistance	Pickleworm	Resistance to Pickleworm (*Diaphania nitidalis*)	PKW
41	Insect resistance	Thrips	Resistance to Thrips (*Thrips palmi*)	THR
42	Insect resistance	Whiteflies	Resistance to Whiteflies (*Bemisia tabaci*)	WFL
43	Vegetative organ	Hypocotyl	Hypocotyl Length	HL
44	Vegetative organ	Cotyledon	Cotyledon Area (size)	CA
45	Vegetative organ	Cotyledon	Cotyledon Length	CL
46	Vegetative organ	Cotyledon	Cotyledon Width	CW
47	Vegetative organ	Leaf	Leaf Bitterness	LB
48	Vegetative organ	Leaf	Leaf Apex-Terminal-Lobe Angle	LAA
49	Vegetative organ	Leaf	Leaf Area (size)	LA
50	Vegetative organ	Leaf	Leaf Attitude	LAT
51	Vegetative organ	Leaf	Leaf Blade Length (base to apex)	LBL
52	Vegetative organ	Leaf	Leaf Blade Width	LBW
53	Vegetative organ	Leaf	Leaf Margin Dentation	LMD
54	Vegetative organ	Leaf	Leaf Margin Undulation	LMU
55	Vegetative organ	Leaf	Leaf Petiole Length	LPL
56	Vegetative organ	Leaf	Trichomes (Vestiture)	TRI
57	Vegetative organ	Vine	Internode Length	IL
58	Vegetative organ	Vine	Node Number (total)	NN
59	Vegetative organ	Vine	Vine Length (plant height)	VL
60	Vegetative organ	Branch	Lateral Branches Number (primary)	LBN
61	Vegetative organ	Root	Root Length (primary)	RL
62	Vegetative organ	Root	Root Number (primary)	RN
63	Vegetative organ	Root	Root Weight (biomass)	RW
64	Vegetative organ	Plant	Biomass (whole plant dry weight)	BIO
65	Flower	Flowering time	(First) Female Flowering Time	FFT
66	Flower	Flowering time	First Flower Node (Position)	FFN
67	Flower	Flowering time	(First) Male Flowering Time	MFT
68	Flower	Flowering time	Flowering Time (days to anthesis)	FT
69	Flower	Sex expression	Female Flower Positions (on main stem and branches)	FFP
70	Flower	Sex expression	Multiple Pistillate Flowers (per node)	MPF
71	Flower	Sex expression	Percentage of Female Flowers (on main stem)	PFF
72	Flower	Sex expression	Percentage of Male Flowers (on main stem)	PMF
73	Flower	Sex expression	Sub-gynoecious	SGY
74	Fruit	Fruit setting	Parthenocarpy (fruit set)	PAR
75	Fruit	Fruit setting	Fruit Setting Positions (# fruits on main stem and branches)	FSP
76	Fruit	Fruit number	Fruit Number (per plant at harvest)	FN
77	Fruit	Fruit growth rate	Fruit Growth Rate	FGR
78	Fruit	Epidermal feature	Fruit Creasing	FCR
79	Fruit	Epidermal feature	Fruit Ribbing	FRB
80	Fruit	Epidermal feature	Fruit Striping (number and length)	FST
81	Fruit	Epidermal feature	Fruit Skin Netting (reticulation)	FSN
82	Fruit	Epidermal feature	Fruit Skin Wax (Glaucosity)	FSW
83	Fruit	Epidermal feature	Fruit Skin Glossiness	FSG
84	Fruit	Epidermal feature	Fruit Skin Mottling	FSM
85	Fruit	Epidermal feature	Fruit Ground Color (commercial fruit stage)	FGC
86	Fruit	Epidermal feature	Fruit Ground Color-Mature	FGCM
87	Fruit	Epidermal feature	Fruit Spine Color	FSC
88	Fruit	Epidermal feature	Fruit Spine Density	FSD
89	Fruit	Epidermal feature	Fruit Spine Size	FSS
90	Fruit	Epidermal feature	Fruit Wart Density	FWD
91	Fruit	Epidermal feature	Fruit Wart Size	FWS
92	Fruit	Shape/Size	Ovary Diameter	OD
93	Fruit	Shape/Size	Ovary Length	OL
94	Fruit	Shape/Size	Ovary Shape Index	OSI
95	Fruit	Shape/Size	Fruit Diameter (Commercial Stage)	FD
96	Fruit	Shape/Size	Fruit Length (Commercial Stage)	FL
97	Fruit	Shape/Size	Fruit Shape Index (Commercial Stage)	FSI
98	Fruit	Shape/Size	Mature Fruit Diameter	MFD
99	Fruit	Shape/Size	Mature Fruit Length	MFL
100	Fruit	Shape/Size	Mature Fruit Shape Index	MFSI
101	Fruit	Shape/Size	Fruit Size (consensus QTL)	FS
102	Fruit	Shape/Size	Fruit Stem End	FSE
103	Fruit	Shape/Size	Fruit Blossom End	FBE
104	Fruit	Shape/Size	Fruit Neck Length	FNL
105	Fruit	Shape/Size	Fruit Hollowness	FH
106	Fruit	Shape/Size	Fruit Weight	FW
107	Fruit	Biomass	Fruit Dry Matter	FDM
108	Fruit	Peduncle	Fruit Peduncle Direction	FPD
109	Fruit	Peduncle	Fruit Peduncle Length	FPL
110	Fruit	Flesh	Flesh Bitterness	FBI
111	Fruit	Flesh	Flesh Color	FLC
112	Fruit	Flesh	Fruit Firmness	FFI
113	Fruit	Flesh	Fruit Flesh Thickness	FTH
114	Fruit	Flesh	Seed Cavity Size	SCS
115	Fruit	Taste quality	Acerbity	ACE
116	Fruit	Taste quality	Acidity	ACI
117	Fruit	Taste quality	Fructose	FRU
118	Fruit	Taste quality	Fruit Water Content	FWC
119	Fruit	Taste quality	Glucose	GLU
120	Fruit	Taste quality	Sucrose	SUC
121	Fruit	Taste quality	Total Soluble Solids	TSS
122	Fruit	Maturity	Fruit Abscission	FAB
123	Fruit	Shelf life	Fruit Shelf Life	FSL
124	Seed	Seed dormancy	Seed Dormancy	SD
125	Seed	Seed number	Seed Number (per fruit)	SN
126	Seed	Seed size	Seed Length	SDL
127	Seed	Seed size	Seed Size	SDS
128	Seed	Seed size	Seed Width	SW
129	Seed	Seed weight	100-Seed Weight	100SW
130	MISC	MISC	Regeneration ability (on MS medium)	RA

Thus, *par6.2* is the second QTL of *parthenocarpic fruit set* on Chr6 (more parthenocarpic fruit is recessive); *Pm1.1* is the first QTL of *powdery mildew resistance* on Chr1 (resistance is dominant); *FS5.3* is the third consensus *fruit size* QTL on Chr5, and *fsd6.2* is the second QTL for *fruit spine density* on Chr6. These rules will be applied in the following discussions for all QTL described but original names are also included for clarity.

For convenience, the 130 quantitative traits were classified into eight categories: Vegetative organ (22), Flower (9), Fruit (50), Seed (6), Abiotic stress tolerance (7), Disease resistance (26), Insect resistance (9), and Miscellaneous (MISC) (1). Under each category, there are also subcategories based on specific plant organs, pathogens, or abiotic stresses (Table 3). In the following sections, under each category, we will briefly discuss selected simply inherited genes and QTL for phenotypic characteristics and their potential in cucumber breeding. Many genes and QTL have a long history of research, but only the most recent literature was cited in the text to save space. The complete list of genes/QTL and references is provided in three supplemental files (1, 2 and 3). For many genes and major-effect QTL, readers can also consult the 2016 Cucumber Gene Catalog^1^ for complete historical references.

## Genes and QTL for whole plant vegetative growth and development

### Simply inherited genes for mutants of vegetative organs

Due to the ease of identification, mutants for foliage characteristics and plant architecture traits such as leaf shape, size, color, and plant height or vine length are frequently reported. Genes responsible for eight cucumber leaf mutants have been identified. The five leaf color mutants are *yp* (*yellow plant*)^6^, *v-1* (*virescent leaf-1*)^7^, *vl* (*variegated leaf*)^8^*, vyl* (*virescent yellow leaf*)^9^, and *Psm* (*Paternal sorting of mitochondria*)^10^. These mutations show a range of phenotypes. The *yp* plant exhibits golden yellow color throughput its life. In the *v-1* mutant, the cotyledons and first 2-3 true leaves are light yellow that turn to green when fully expanded; subsequent true leaves are green from the beginning. The young leaves on the *vyl* mutant are yellow and gradually turn green when mature, whereas all leaves of the *vl* mutant show a green and light yellow/white variegation which is especially obvious on younger leaves. All these mutants show some degree of retarded growth and reduced vine length, but the fertility and fruit set seem unaffected. The *Yp* gene (*CsCHLI*) is a homolog of the gene for the Mg chelatase I subunit; Mg chelatase is a rate-limiting enzyme in the chlorophyll biosynthesis pathway. The candidate gene for *Vyl* is predicted to encode a DnaJ-like zinc finger protein involved in regulation of chloroplast development, whereas *v-1* seems to encode a cyclic-nucleotide-gated ion channel protein (*CsCGNC*). The nuclear *pentatricopeptide repeat 336* gene (*CsPPR336*) is the candidate for the *Psm* locus underlying paternally transmitted mosaic phenotypes^10^.

Wild type cucumber leaves are flat and have seven lobes with toothed or smooth margin. Three non-lobe, *round leaf* mutants, *rl-1*, *rl-2* and *rl* have been identified, which are all due to allelic mutations in the *PINOID* (*CsPID*) gene encoding a regulator for the auxin polar transporter PIN (PIN-FORMED)^11,12^.

The leaf margin of the two *curly leaf* mutants, *cul-1* and *cul-2* rolls upward forming a shallow cup; both mutants are due to allelic mutations in the *CsPHB* gene for a class III homeodomain-leucine zipper (HD-ZIP III) transcription factor^13^. The *tendrilless* (*ten*) mutation is caused by a SNP in the *TEN* gene encoding a TCP transcription factor^14^. Another *tendrilless-1* (*td-1*) mutation has been mapped to a ~190 kb region in Chr6 (ref. ^15^). The phenotypes of the two *tendrilless* mutants are very different; *ten* is phenotypically normal except that the ‘tendril’ develops into leaves with long petioles and thin branches, whereas *td-1* mutation has more widespread pleiotropic effects.

The *littleleaf* (*ll*) mutant, which produces leaves approximately one quarter of the size of standard American pickling cucumbers, was identified ~40 years ago. *LL* is a homolog of Arabidopsis *STERILE APETALA* (*CsSAP*) encoding a WD40 repeat domain-containing protein^16^. QTL analysis revealed co-localization of major-effect QTL for fruit size, fruit weight, seed weight, and multiple lateral branches with the *LL* locus indicating pleiotropic effects of the *ll* mutation. In addition, *ll* cucumbers often have poor internal fruit quality, which may hinder its use in pickling cucumber breeding.

Plant architecture, especially plant height or vine length, is important in cucumber breeding. So far, six mutants with reduced internode length or compact growth habit have been reported including *compact* (*cp*)^17^, *compact-1*(*cp-1*)^18^, *short internode* (*si*)^19^, *super compact-1*(*scp-1*)^20^, *super compact-2* (*scp-2*)^21^, and *dwarf* (*dw*)^22^. The *cp-1*, *dw*, *scp-1*, and *scp-2* mutants have extremely short internodes with little value in practical use. Both *scp-1* and *scp-2* are due to mutations of genes in the brassinosteroid (BR) biosynthesis pathway including *CsCYP85A* for the BR-C6-oxidase, and *CsDET2* for the steroid 5-alpha-reductase^20,21^. The *si* mutant exhibits short internode (~50% of WT) and small fruit, which is a homolog for the gene encoding a member of the VIER F-BOX PROTEIN subfamily of the F-Box protein family (*CsVFB1*)^19^.

Hypocotyl elongation of modern commercial cucumbers is sensitive to environmental conditions. For example, high temperature or low light intensity may increase hypocotyl length resulting in poor seedling quality for transplanting. The semi-wild Xishuangbanna (*C.s*. var. *xishuangbannesis*, XIS) and wild (*C.s*. var. *hardwickii*, HARD) cucumber populations are enriched with the *short hypocotl1* (*sh1*) allele, which renders hypocotyl elongation insensitive to UVB-free light and temperature changes^23^. *Sh1* (*CsSH1*) is a homolog of the gene encoding a human SMARCA3-like chromatin remodeling factor. The *sh1* mutation may be of value in use for mass seedling production in protected environments.

Four glabrous (trichome-free) mutants have been reported. The “*glabrous1*” (*csgl1*) or “*micro-trichome*”(*mict*) mutant shows no visible trichomes on all aerial organs except the hypocotyl. *CsGL1* encodes a Class I HD-ZIP transcription factor^24,25^. The *csgl2* mutant exhibits glabrous stem, petioles, and leaves, but fruit, sepals, fruit peduncles, and flower pedicel are covered with sparse and fine hairs, and the candidate gene for this mutation is unknown^26^. The *csgl3* (*tril*) mutant is completely free from trichomes which encodes a Class IV HD-ZIP transcription factor; the glabrous phenotype in *csgl3* is due to either SNPs or retrotransposon insertion in the coding region^27–29^. The *csgl4* mutant has glabrous fruit skin but reduced size and number of trichomes on the stem and leaves; *CsGL4* was thought to encode a C-type lectin receptor-like tyrosine-protein kinase^2^.

### QTL for vegetative growth and development-related traits

**Fig. 3:**
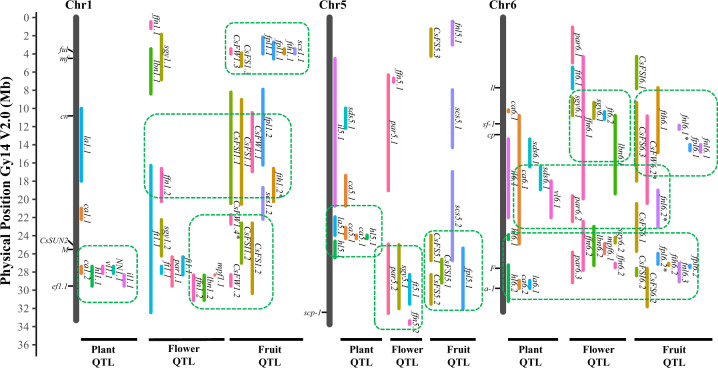
Chromosomal locations of vegetable organ-, flower- and fruit set-related QTL on cucumber chromosomes 1, 5, and 6. Ruler to the left indicates locations (in Mbp) in the Gy14 V2.0 draft genome assembly (drawn to scale). Vertical black lines are chromosomes. Cloned genes and QTL are aligned to the left and right of each chromosome, respectively. Vertical bar for each QTL represents 1.5 or 2.0 LOD confidence interval on the chromosome. Dashed rectangles indicate gene/QTL hot spots or clusters. CA = cotyledon area, CsFS = consensus fruit size and shape, FFN = first flower node, FT = flowering time, FPL = fruit peduncle length, FW = fruit weight, HL = hypocotyl length, IL = internode length, LA = leaf area, LBN = lateral branch number, MPF = multiple pistillate flowers, NN = node number, SCS = seed cavity size, SDS = seed size. SGY = sub-gynoecious, PAR = Parthenocarpy, and VL = vine length.

Significant variation exists among cucumbers for size of vegetative organs such as hypocotyl length (HL), cotyledon area (CA), leaf area (LA), vine length (VL), internode length (IL), total number of nodes (NN), lateral branch number (LBN), and whole plant (above-ground) biomass (BIO). QTL mapping studies for these traits were conducted primarily using three RIL populations from the following crosses: S94 × S06, 9110Gt × 9930, and PI 183967 × 931 (ref. ^30–34^). Details of all detected QTL for these traits and their chromosomal locations are presented in Fig. 3, and Supplementary Files 3 (Table S3) and 4 (Fig. S1).

Six moderate-effect (PVE ~10%) and one (*hl6.2*) large-effect (PVE = 22.6%)^32,34^ QTL, were identified for HL in two RIL populations, but none are co-localized with *sh1*. QTL mapping on cotyledon/leaf length and width was conducted in two RIL populations^32–34^. Eight CA (cotyledon area) and five LA (leaf area) consensus QTL from these studies are listed in Supplementary File 3, of which three LA and CA QTL were co-localized suggesting possible shared mechanisms in regulation of cotyledon and leaf sizes in the two populations.

In the 9110Gt × 9930 RIL population, 7 QTL for plant architecture-related traits were detected including four for IL, one for NN, and two for VL. Given the role of node number and internode length on vine length, the 3 major-effect QTL (*il1.1*, *nn1.1*, and *vl1.1*) are co-localized on Chr1 (Fig. 3). The number of lateral branches (LBN) varies significantly in different cucumbers. The *littleleaf* (*ll*) mutant H19 also has multiple lateral branches, which is likely due to the pleiotropic effect at the *ll* locus^16^. In the S94 × S06 RIL population, there were 6 QTL underlying LBN variation including two major-effect QTL (*lbn1.2*, and *lbn6.2*), but none is located nearby the *ll* locus indicating multiple mechanisms regulating branch numbers.

The observed clustering of these size- or length-related QTL for vegetative organs (Fig. 3; Supplementary File 4) on four chromosomal locations on Chr1, Chr5, and Chr6, suggest common genetic basis for these traits.

## Genes and QTL for reproductive development

### Simply inherited genes for sex determination

A cucumber plant can bear male, female, or bisexual flowers, and their combinations result in five major sex morphs: monoecious (male and female flowers), andromonoecious (male and perfect flowers), gynoecious (female only), androecious (male only) and hermaphroditic (bisexual flowers only). In cucumber, sex determination depends primarily on the *F* (*femaleness*), *m* (*andromonoecy*), and *a* (*androecy*) loci, all of which are members of the *aminocyclopropane-1-carboxylic acid synthase* (*ACS*) gene families (*CsACS1* for *F*; *CsACS2* for *M*, and *CsACS11* for *A*) catalyzing the rate-limiting step in ethylene biosynthesis^35–37^. The *F* locus is consisted of two copies of *ACS1* (*CsACS1* and *CsACS1G*). Additional genes or modifiers affecting sex expression also exist. For example, mutations in *CsACO2* (*a-1*) for the 1-aminocyclopropane-1-carboxylate oxidase result in androecy^38^. A major-effect QTL, *Sgy3.1*, controls *F* locus-independent high percentage of female flowers on monoecious plants^39,40^(also see below). These diverse sex-determination genes provide opportunities to fine tune sex expression for cucumber production.

### QTL for reproductive development-related traits

#### Flower- and fruit set-related QTL

Flowering time (FT) and sex expression are directly related to fruit timing and yield, respectively. The wild cucumber (*C.s*. var. *hardwickii*), semi-wild XIS cucumber, and some landraces from India and Pakistan require short-day length for flower induction. For example, it takes six or more months for the XIS cucumber accession WI7167 to flower under long-day conditions^41^, while most modern varieties will flower in 30–50 d after planting. In two studies, four QTL (*ft1.1*, *ft5.1*, *ft6.1*, and *ft6.2*) were found to control flowering time variation in populations derived from two XIS cucumber accessions (SWCC8 and WI7167)^41,42^ (Supplementary File 5 or Fig. S2). Two other studies used populations derived from crosses between cultivated cucumber lines with <5d FT difference. In each case, a single major-effect FT QTL (*da1.1* and *Ef1.1*) was detected^32,43^; both are very close to *ft1.1*. It was suggested that *ft6.2* in WI7167 is a major-effect QTL regulating day-length sensitive flowering while *ft1.1* regulates flowering time within cultivated cucumbers^41^.

Early fruit yield is influenced by flowering time and position of the first flower node (FFN). Nine FFN QTL were identified in two monoecious (*ff*) × gynoecious (*FF*) RIL populations^30,32,44^. Among three major-effect FFN QTL, *ffn6.2* was located near the *F* locus as expected, while QTL *ffn1.2* and *ffn3.2* also have major effects (Fig. 3). These studies revealed the complicity of genetic control of the FFN trait, which is obviously the results of the interplay among factors affecting both flowering time and sex expression.

A gynoecious plant carrying the homozygous *FF* gene has one or more female flowers on each node, which may not be ideal in some production systems with less optimal cultural practices or poor production conditions since not all female flowers will develop into marketable fruit. The term “sub-gynoecious” (SubG) type sex expression was used to describe the plant that starts with male flowers in the first 5–10 nodes and then has continuous female flowers on the main stem with an overall percentage of female flowers (PFF) of >80% (ref. ^39^). In a segregating population derived from the cross between S-2-98 (SubG) and M95 (M), 4 QTL, *Sgy3.1*, *Sgy4.1*, *Sgy6.1*, and *Sgy6.1*, were found to regulate PFF with *Sgy3.1* having the strongest effect (PVE = 54.6%)^39^. In another study, Win et al. confirmed the major-effect QTL *Sgy3.1*, and identified two additional QTL, *Sgy1.1* and *Sgy1.2*, which are able to increase, and decrease PFF, respectively^40^ (Supplementary File 3). A gene for the GA20-oxidase was proposed to be the candidate gene for the dominantly inherited *Sgy3.1* locus^40^. Phenotypically, an *F* gene-independent SubG plant is similar to the one that is heterozygous at the *F* locus (*Ff*), which usually starts with male flowers in the first few nodes (1–10) followed by continuous female flowers. When QTL mapping for PFF was conducted using populations derived from gynoecious (*FF*) × monoecious (*ff*) crosses^44,45^, as expected, the major-effect QTL for PFF was consistent with the *F* locus (Fig. 3). Minor-effect PFF QTL were detected in these studies, which seem to co-localize with SubG QTL *Sgy3.1* and *Sgy6.1*^39,40^ (Supplementary File 5). These observations suggest the PFF is influenced by multiple genetic factors although the *F* and *Sgy3.1* loci play the major roles in gynoecious and SubG plants, respectively.

Some gynoecious cucumber lines may bear multiple pistillate flowers (MPFs) at each node. Five MPF QTL have been identified with each having similar effect (PVE ~10%)^46^. Parthenocarpic fruit set (PAR) is critical for cucumber production in protected environments. Lietzow et al. and Wu et al. detected 12 PAR QTL in two sources, but only two (*par2.1* and *par7.1*) are co-localized between the two studies^47,48^. The inconsistent results reflect the difficulties in accurate phenotyping for PAR, which is difficult to separate from yield.

Many of the FT- and sex-expression-related traits are correlated and may be regulated by common, hormone-related pathways, which can be evidenced from QTL clusters for different traits on chromosomes 1, 3, 5, 6, and 7 (Fig. 3; Supplementary File 5).

## Genes and QTL for fruit–related traits

### Genes for simply inherited fruit-related traits

#### Fruit skin and flesh color

Cucumber fruit exhibits a wide spectrum of skin colors that can vary from light green, yellow green, green, dark green, to creamy, white, yellow, brown, orange, or red (Fig. 1). The *white skin color* (*w*) is due to a mutation in the *CsAPRR2* gene, which plays an important role in fruit pigment accumulation^49^. Mutations in the *lgp* (*light green peel*, *CsARC5*) and *lgf* (*light green fruit*, *CsYcf54*) genes cause change of dark green fruit color to light green^50,51^. The *orange/red mature fruit color* locus *R* is allelic to the *black spine* gene *B*, which encodes a R2R3 MYB transcription factor^52,53^.

Most cucumber fruits have white flesh. The semi-wild XIS cucumber has *orange flesh* (*or*) and accumulates high-level β-carotene at mature fruit stage. This is due to a mutation in *CsBCH* for β-carotene hydroxylase^33^. The *yellow flesh* (*yf*) locus from PI 200815 was fine mapped into a 150-kb region on Chr7 (ref. ^54^). The *green flesh* (*gf*) in immature cucumber, results of accumulation of chlorophyll, is controlled by two loci^55^.

#### Fruit epidermal features

The external appearance of cucumber fruit is important for consumer acceptance or processing. Several simply inherited genes determine fruit epidermal features, some of which are tightly linked on Chr5 (Fig. 2) including *Heavy/no netting* (*H/h*), *Warty/smooth fruit* (*Tu/tu*), *Dull/glossy fruit skin* (*D/d*), *Ribbed/non-ribbing fruit* (*Fr/fr*), *Mottled/uniform immature fruit color* (*U/u*), *Large/small spines* (*SS/ss*), and *Tough/tender fruit* (*Te/te*). Interestingly, specific allele combinations of these genes are characteristic of different market classes. For example, the European Long, Chinese Long, and US pickling cucumbers often have *u-H-tu-ss-te-fr-d*, *u-h-Tu-ss-te-Fr-d*, and *U-h-Tu-SS-Te-fr-D* haplotypes, respectively. This is likely the result of diversifying selection during breeding for different market classes.

The number of spines on the fruit vary widely in cucumbers of different market classes. The *few spine1* (*fs1*) mutation identified from a dense-spined Chinese Long line is due to an 812-bp deletion in the promoter region of *CsGL3* (ref. ^56^); but higher density spines in Chinese Long cucumber seem to require both *CsGL3* and the QTL *fsd6.1* (ref. ^57^). Some cucumbers have *numerous* (*ns*) but *small spines* (*ss*) with the *ns* being a homolog for the gene encoding an auxin transporter-like protein 3 (*CsLAX3*)^58,59^. Fruit spines usually are hard and prickly and may cause an itching response on the skin. A *tender spine* (*tsp*) mutant does not trigger itching, which seems due to an N-terminal deletion in *Tsp* for a C-type lectin receptor-like tyrosine-protein kinase^3^. A non-synonymous mutation within the same gene was proposed to confer glabrous trait (cs*gl4*) with smaller and fewer trichomes^2^. Fruit spines often sit on a bulge structure of several layers of cells called tubercles (warts). The *Tu* (*tuberculated*) locus controls wart development, and *Ts1* regulates *tubercle size*, which encodes a C_2_H_2_ zinc finger domain-containing transcription factor (*CsTu*), and an oleosin (*CsTs1*), respectively; *CsTu* can bind directly to the promoter of *CsTs1* to promote its expression^60,61^.

### QTL for fruit size/shape, external and internal fruit quality traits

#### Fruit size and shape

Cucumber exhibits diverse fruit size (FS) and fruit shape. Fruit shape is defined using fruit shape index (FSI) which is the ratio of fruit length (FL) to fruit diameter (FD). In some cases, simply inherited genes have been found to play important roles in fruit size control. For example, the *fruitful1* (*CsFUL1*) gene is a key player in fruit elongation in Chinese Long cucumber^62^. Of two *short fruit* mutants (*sf-1* and *sf-2*) recently identified^4,63^, s*f-2* encodes a cucurbit-specific RING-type E3 ligase, which results in its enhanced self-ubiquitination and degradation, as well as increased expression *CsACS2* (*m* locus). This may also explain the elongated fruit due to an allelic mutation of the *m* locus (*m-1*) on an andromonoecious plant (*m-1m-1*); an andromonoecious cucumber plant (*mm*) usually sets round fruit^64^. Fruit size variation in cucumber is also influenced by fruit carpel number (CN). CN variation (3 vs 5) is controlled by the *Cn* gene that is a homolog of *CLAVTATA3* (*CsCLV3*)^65^. Cucumber fruit shape can be round, oval, oblong, long or very long. A spontaneous mutant bears mango-shaped fruit (*mango fruit, mf*) which is due to a SNP in the *WUSCHEL-related homeobox1* (*CsWOX1*) gene^66^.

In most cases, fruit size and shape are controlled by QTL. A number of QTL mapping studies on fruit size/shape have been conducted in cucumber. Pan et al. reviewed the genetic architecture of fruit size variation in cucumber, and identified 19 consensus fruit size (FS) and 11 fruit shape (FSI) QTL^67^. Among them, the consensus FS QTL *FS1.2* and *FS2.1* are the homologs of tomato *SUN (CsSUN2*) and *SlTRM5* (TONNEAU1 Recruiting Motif) (*CsTRM4*), respectively^67–69^. Details of these consensus FS QTL are presented in Supplementary Files 3 and 6. In addition, fruit weight (FW) is apparently correlated with fruit size, which is also an important component for fruit yield. QTL mapping have identified 19 FW QTL in three studies^42,44,70^(Supplemental Files 3 and 6). Almost all FW QTL are co-localized with consensus FS QTL indicating a close correlation between them.

Two other traits often correlated with fruit length are fruit neck length (FNL) and fruit peduncle length (FPL). Fruit neck is the stem-end of the fruit with reduced fruit expansion, which usually does not have spines. Long fruit neck is an undesirable trait because it gives non-uniform external appearance and often has a bitter taste due to accumulation of cucurbitacins. FNL is strongly associated with fruit length. In the only QTL mapping study for FNL^44^, all five QTL were co-localized with the FS consensus QTL (Supplementary File 6). Fruit peduncle connects the stem and the fruit. There is significant variation in FPL among different cucumber market classes. Seven FPL QTL were identified in two studies^44,71^; all of which are co-localized with FS consensus QTL.

A fruit with small seed cavity and thick flesh is preferred for both processing and fresh market uses. Structurally, fruit seed cavity size (SCS) and fruit flesh thickness (FTH) are two traits to describe the endocarp and mesocarp of the cucumber pepo fruit, respectively. Eight and six consensus QTL have been identified for SCS and FTH, respectively^44,72^.

As discussed earlier, most fruit epidermal feature genes are simply inherited (Tables 1 and 2), but some show quantitative variation. For example, Tian et al. found that fruit skin wax (glaucosity) (FSW) accumulation is controlled by five QTL, with *fsw5.1*, and *fsw6.1* having moderate effects^73^ (Supplementary File 3). Shimomura et al. and Miao et al. examined fruit wart size (FWS) and density (FWD) and identified 3 and 2 QTL, respectively^74,75^. In both cases, the major-effect QTL is consistent with the *Tu* locus (Table 1). Fruit spine density on cucumber fruit may vary from very few large spines, many *small spines* (*ss*), to high-density spines or ultra-high-density hairs (or *numerous spines*, *ns*). The *ns* and *ss* single genes have been cloned or fine mapped (Tables 1 and 2). Bo et al. examined spine density in bi-parental and natural populations, and identified three QTL: *fsd6.2*, *fsd6.1*, and *fsd4.1* that control high and ultra-high spine densities, which had major-, moderate, and minor effects, respectively^57^. The *fsd6.2* locus, which is a variant of the *CsGL3* gene (Table 1) regulates high spine density, but for ultra-high spine density, both *fsd6.1*, and *fsd6.2* are required.

## QTL for seed-related traits

Cucumber seed did not seem to be a target of selection during long-term cultivation. Cucumber seeds are white or gray in color, but seed size does show significant variation especially between the wild and cultivated cucumbers. The wild cucumber accession PI 183967 has very small seeds. In two studied, Wang et al. and Lietzow conducted QTL analysis for seed length, width, and weight^76,77^. Most QTL for the three traits are co-localized, and the seven consensus QTL for seed size (SDS) are summarized in Supplementary File 3. Seed size did not seem to have any obvious correlation with other size or length-related traits (Supplementary File 6).

## Genes and QTL for disease resistances and abiotic stress tolerances

### Genes for simply inherited disease resistances

Major cucumber diseases of worldwide importance include downy mildew (DM), powdery mildew (PM), angular leaf spot (ALS), target leaf spot (TLS), anthracnose (AR), Fusarium wilt (FOC), scab, and various viruses like cucumber mosaic virus (CMV), watermelon mosaic virus (WMV), zucchini yellow mosaic virus (ZYMV), and papaya ringspot virus (PRSV). The cucumber accession PI 197087 from India and its derivatives like Gy14 are resistant to DM, ALS and AR that is conferred by *dm1*, *psl*, and *cla*, respectively. It was found that the cucumber *Staygreen* (*CsSGR*) is the causal gene underlying the *dm/psl/cla* locus (Chr5 in Fig. 2); thus, the durable resistance against the three different pathogens (bacterial, oomycete, and fungal) in Gy14 is due to a loss-of-susceptibility mutation in *CsSGR*, which encodes the Mg dechelatase that plays critical regulatory roles in the chlorophyll degradation pathway^78,79^. The *dm1*-conferred DM resistance was less effective since 2004 when new DM pathogen strains emerged in the cucumber field in the US. Two major-effect QTL for resistance against the post-2004 DM strain(s) (*dm4.1* and *dm5.2*) were identified from PI 197088 and PI 330628 (ref. ^80,81^).

Another well characterized loss-of-susceptibility *R* gene in cucumber is the *mlo* locus for PM resistance^82–84^. Multiple allelic variants at this locus have been identified in PM resistant accessions; all result in the loss of function of *CsMLO*. Additional PM resistance genes near the *mlo* locus are also possible^85^ (Table 2, Fig. 2). The Chinese Long line, Jin5-508, carries a dominantly inherited PM resistance gene *Pm1.1* which has been mapped in a 41.1-kb region containing two cysteine-rich receptor-like protein kinase genes^86^.

Three recessively inherited TLS resistance genes, *cca-1, cca-2, cca-3*, have bene mapped on Chr6 (Fig. 2)^87,88^. Among them, *cca-3* seems to belong to the CC-NB-ARC type *R* gene family^88^ which has ~73 homologs in the cucumber genome. In addition, the closely linked *ccu* for scab resistance and *Foc* for *Fusarium* wilt resistance were mapped to a region on Chr2 containing a cluster of several NB-LRR *R* gene homologs^89,90^.

The candidate gene for the *zym* locus (*CsVPS4*) for ZYMV resistance encodes the vacuolar protein sorting-associated protein 4 (VPS4)-like protein^91^. Several variants of the *zym* locus have been identified in different ZYMV resistance sources^92^. Three virus resistance genes have been mapped on Chr6 including *prsv* for PRSV*, wmv* for WMV and *cmv6.1* for CMV^93–95^. Previous studies indicated tight linkage of resistances to three potyviruses (PRSV, ZYMV and WMV) in cucumber. Molecular mapping results seem to suggest that they are different loci (Fig. 2).

### QTL for disease resistances and abiotic stress tolerance

**Fig. 4:**
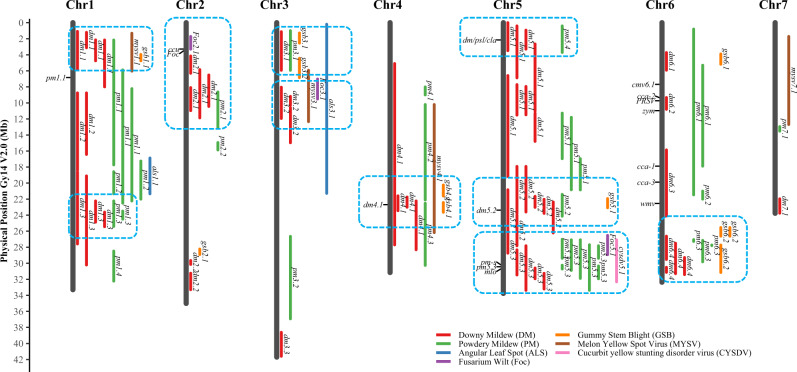
Chromosomal locations of disease resistance genes and QTL in cucumber. Ruler to the left indicates locations (in Mbp) in the Gy14 V2.0 draft genome assembly (drawn to scale). Vertical black lines are chromosomes. Cloned genes and QTL are aligned to the left and right of each chromosome, respectively. Vertical bar for each QTL represents 1.5 or 2.0 LOD confidence interval on the chromosome. Regions delimited by blue dashed rectangles indicate resistance gene/QTL hot spots or clusters.

QTL studies have been carried out for resistances to the following diseases: PM, DM. FOC, Gummy stem blight (GSB), Melon Yellow Spot Virus (MYSV), and the Cucurbit Yellow Stunting Disorder Virus (CYSDV). The results are summarized in Table 4, and their chromosomal locations are illustrated in Fig. 4. More details for each QTL are presented in Supplementary File 2 (Table S2).

**Table 4:** Summary of disease resistance QTL identified in cucumber.

Diseases^a^	Resistance Sources	QTL and effects^b^	Notes
**PM**	**PI 197088**	pm1.1**, pm1.3**, pm2.1, pm2.2, pm3.1, pm4.3*,pm5.1**, pm5.3**, pm5.4**, pm6.1**, pm6.3**, pm7.1**	
	**S06** (Beit alpha)	pm1.2, pm4.1**, pm5.1, pm6.3**	
	**WI 2757**	pm1.1**, pm1.2*,pm3.2, pm4.2*, pm5.2**, pm5.3**	
	**H136** (Chinese Long)	*pm1.3, pm6.2*	Detected with BSA
	**K8** (Chinese Long)	pm5.1, pm5.3**,pm6.3	
	**IL52** (*C.hystrix* IL)	pm5.3**	Single gene
**DM**	**WI7120** (PI 330628)	dm2.1,dm4.1**,dm5.2**,dm6.3,dm6.4	
	**IL52, CCMC** (Chinese Long)	dm1.1, dm1.2, dm1.3, dm5.1**, dm5.3**, dm6.4	Three Chr1 QTL from CCMC
	**K8**	dm1.1**, dm5.2**, dm6.4	
	**PI 197088**	dm1.1, dm1.2,dm1.3*, dm2.1,dm2.2, dm3.1, dm3.2, dm3.3, dm4.1**, dm5.1**, dm5.2**, dm5.3**, dm6.1,dm6.2,dm6.4, dm7.1	
	**PI 197085**	dm5.1*, dm5.2*, dm5.3*	
	**S94**	dm1.1**, dm5.1**	
	**TH118FLM**	dm2.1**, dm2.2**, dm5.1**	Drived from ‘Malini' F1 hybrid
	**WI2757**	dm1**, dm5.2**	
**ALS**	**WI2757**	psl**,als1.1, als3.1	
**FOC**	**9110Gt** (European Long)	Foc2.1**	Single gene
	**URS189**	*Foc3.1, Foc5.1*	Patent
**GSB**	**PI 183967** (wild cucumber**)**	gsb1.1, gsb2.1, gsb6.1**, gsb6.2	Mature pant resistance
	**PI 183967**	gsb3.1, gsb3.2, gsb4.1, gsb5.1**, gsb6.2	Seedling stage resistance
	**HH1-8-1-2** (Chinese Long)	*gsb4.1, gsb6.2*	Seedling stage resistance
**CYSDV**	**PI 250147**	*cysdv5.1*	Single gene
**MYSV**	**Tokiwa**	mysv1.1**, mysv3.1**, mysv4.1*, mysv7.1	Resistance to spotted wilt

^a^Complete references are provided in Supplementary File 1 (Table S1)

^b^*PVE (percentage of phenotypic variance explained) = 10–15%; ** PVE > 15%; underlined: contribute to disease susceptibility

QTL mapping for PM resistance (PMR) has been conducted from six resistance sources including PI 197088 (ref. ^81,96,97^), S06 (ref. ^98^), K8 and H136 (ref. ^99^), WI2757 (ref. ^100^), and IL52 (ref. ^85^). Diverse mapping populations, phenotyping and genotyping methods were used in these studies with varying power of QTL detection. However, based on chromosomal locations of these QTL, 19 consensus PMR QTL could be inferred (Supplementary File 2). The co-localization of QTL from different resistance sources may suggest that they belong to the same locus, or are closely linked. For example, *pm5.3* was detected in PI 197088, IL52, WI 2757 and K8; both *pm5*.1 and *pm6.3* were detected in S06, K8 and PI 197088. The *pm5.3* locus (syn. *pm5.1*, *pm-h*) encodes a barley *MLO* homolog (*CsMLO1*), and multiple variants at this locus are responsible for PMR in different lines^82–84^. The *pm*/*dm5*.3 QTL has been shown to confer complete PM resistance in IL52, and the gene for a GATA transcriptional factor was proposed to be its candidate^85^.

QTL mapping for DM resistance (DMR) has been conducted in PI 197085, PI 197088, WI 7120 (PI 330628), WI 2757, S94, TH118FLM, IL52, and K8 (Table 4). Sixteen QTL were identified in PI 197088, and four of them are major-effect QTL contributing to DMR (*dm4.1*, *dm5.1*, *dm5.2*, and *dm5.3*)^81,101,102^. PI 330628 carries five DMR contributing QTL with *dm4.1* and *dm5.2* having the largest effect^80^. WI 2757 exhibits moderate resistance to post-2004 field DM strains and carries both *dm1* from PI 197087 and *dm5.2* with unknown origin^81^. Among the 17 consensus DMR QTL, 11 could be detected in at least two resistance sources (Table 4; Supplementary File 2). Interestingly, the two major-effect QTL, *dm5.1* and *dm5.2* were detected in five resistance sources, whereas *dm1.1* and *dm6.4* were each identified in four lines. These observations suggest that cucumbers from different origins may share some comment genetic basis for DMR although the magnitude of these QTL are affected by genetic backgrounds and environmental conditions.

QTL mapping studies for resistances to other pathogens are sporadic. *Fusarium* wilt is a soil-borne disease, which is more serious in cucumber production under protected environments. A major-effect QTL for Fusarium wilt resistance, *Foc2.1* was identified, which is closely linked with the scab resistance (*ccu*) locus in a region with multiple members of NB-LRR resistance gene homologs^89,90,103^. The wild cucumber line PI 183967 is highly resistant to GSB. The adult plant and seedling GSB resistances were controlled by four and five QTL, respectively^104,105^, but only one minor-effect QTL (*gsb6.2*) is shared between the two stages. Two minor-effect GSB resistance QTL (*gsb4.1*, and *gsb6.2*) were also detected in a *C. hystrix* introgression line^106^. For virus resistances, four QTL for the resistance to isolate MYSV-FuCu05P-2 have been identified^107^. A major-effect QTL for CYSDV resistance (*cysdv5.1*) was mapped to a region close to the *mlo* locus for PMR^108^.

In cucumber breeding, it has long been observed that there is a positive correlation between resistances to different pathogens such as DMR and PMR, resistance to Fusarium wilt and scab, and resistance to different potyviruses (e.g., PRSV, and ZYMV). Indeed, several lines used in the above-mentioned QTL mapping studies possess dual resistances to PM and DM (for example, PI 197088, K8, IL52, and WI2757). The chromosomal locations of consensus resistance QTL to different pathogens are illustrated in Fig. 3. Clearly, many disease resistance QTL are co-localized, which is especially true for PM and DM. Also, it seems there are several hot spots on chromosomes 5 and 6 where resistance loci to different pathogens are highly enriched (Fig. 3). This offers potential advantages in disease resistance breeding for cucumber. However, at the molecular level, whether these resistance genes or QTL belong to the same locus, or are closely linked await further investigation.

Cucumber is of tropical origin and is sensitive to low temperature. In temperate growing regions or production areas at a high altitude, low temperature germination (LTG) ability is a trait that may allow for early planting. In two studies^109,110^, four LTG QTL were identified: *LTG1.1, LTG1.2, LTG2.1*, and *LTG4.1*. The two major-effect contributing QTL, *LTG1.1* and *LTG1.2*, are 2-Mbp apart on Chr1 (Supplementary File 3). Waterlogging is a serious environmental stress in many cucumber production regions. One strategy for cucumber plants to deal with the waterlogging stress is the production of hypocotyl-derived adventitious roots (AR). In the waterlogging resistant line Zaoer-N, three QTL contribute to increasing AR numbers under waterlogging^111^. The gene for an AAA-ATPase domain-containing protein has been shown to be a candidate for the major-effect QTL for *adventitious root numbers*, *ARN6.1*^112^.

## Genes for MISC horticulturally important traits

The bitter tasting cucurbitacins are tetrocylic terpenes present widely in cucurbit crops. Three bitterness related genes have been cloned including *Bi* (*bitterfree*), *Bl* (*bitter leaf*), and *Bt* (*bitter fruit*)^113^. *Bi* encodes a cucurbitadienol synthase that catalyzes the cyclization of 2,3-oxidosqualene into the tetracyclic cucurbitane skeleton, the first committed step of cucurbitacin biosynthesis. Both *Bl* and *Bt* encode two basic helix-loop-helix (bHLH) transcription factors that are expressed specifically in leaves and fruits, respectively. *Bl* binds to the E-box elements of the *Bi* promoter to activate its transcription for cucurbitacin biosynthesis in cucumber leaves; *Bt* has similar biochemical function as *Bl* but regulates cucurbitacin biosynthesis in the fruit^113^. Abiotic stress influences cucurbitacin biosynthesis by modulating the expression of *Bl* and/or *Bt*^113^.

Cucumber foliage or fruit are usually non-fragrant, but some varieties from Thailand have pandan-like fragrance from leaves and fruit, which is controlled by the *fgr* (*fragrance*) locus (*CsBADH*) encoding the betaine aldehyde dehydrogenase^114^.

## Concluding remarks

New genomic technologies and resources for cucumber have allowed for a surge in research leading to QTL mapping and identification of candidate genes associated with a wide array of phenotypic traits. In this work we documented 81 simply inherited genes or major-effect QTL and 322 QTL for 42 quantitative traits, providing chromosome locations, allelic variants and associated polymorphisms, predicted functions where appropriate, and diagnostic markers that could be used for marker-assisted selection in cucumber breeding. Despite the increased effort in cucumber, the number of cloned genes and narrowly defined QTL is still quite limited, and in most cases the proposed functions have not been verified. Looking to the future, it is anticipated that studies in cucumber will be able to draw on an increasing number of genomic tools, both to identify and verify important genes. Cucumber collections in major gene banks are rich in genetic variation that could be explored to identify novel genes or alleles. Genome-wide association analysis may play an important role to accomplish this. EMS mutagenesis is also a powerful tool to generate novel mutations and development of efficient genetic transformation and gene editing systems will allow characterization of gene functions.

It is hoped that the present work will serve as starting point for the systematic inventory of cucumber genes, quantitative trait loci, genetic stocks, and mutants, to benefit the cucurbit community in the years to come. As the information about cucumber genes continues to grow, it has also become imperative for the community to adopt a standard nomenclature to describe QTL. Standardized nomenclature, as has been adopted for numerous other species, facilitates continued progress and minimizes confusion when comparing results across publications. We hope the vocabularies for quantitative traits and the QTL naming rules we recommended here will help achieve this goal.

## Supplementary information

Supplementary files 1 to 7
