# Supplementary material for: Molecularly tagged genes and quantitative trait loci in cucumber with recommendations for QTL nomenclature
Source: Hortic Res. 2020 Jan 1;7:3. doi: 10.1038/s41438-019-0226-3 (PMC6938495; doi:10.1038/s41438-019-0226-3)
Supplement: Supplementary file 1 — Supplementary files 1 to 7 [file 41438_2019_226_MOESM1_ESM.pdf]

Table S1. Details of identified genes or major-effect QTL for simply inherited traits in cucumber in cucumber (as of July 2019).

#	Category	Sub-category	Gene/mutants	Gene	Candidate gene	Variant/haplotype	Predicted functions	Cloned (Y/N)	Mutant Type	Gene (9930 V2.0)	Gene (Gy14 v2.0)	Materials/Lines	Polymorphisms between WT and mutant parental lines	Chr	Position/interval (9930V2.0)	Position (Gy14V2.0)	Diagnostic markers	Marke Type	Forward-Marker	Reverse-Marker	Common primer (for KASPAR marker)	References
1	Vegetative organ	Hypocotyl	*Short hypocotyl1*	*sh1*	*CsSH1*		Homolog of the humanSMARCA3	Y	Natural	*Csa3G141820*	CsGy3G012350.1	SWCC8	SNP (C to T), results in an amino acid substitution (Arg490His)	3	9,286,025	9,318,259	dCAPS-51	dCAPs, RsaI	ATACTTACTCATCACAGGTA	TTCCCTCTTTGTGCGTTACTT		23
2	Vegetative organ	Leaf	*Curly leaf1*	*cul-1*	*CsPHB*	*Haplotype1*	Class III homeodomain-leucine zipper (HD-ZIP III) t	Y	EMS	*Csa6G525430*	CsGy6G036200.1	CCMC^{1561}	SNP (C to T), results in an amino acid substituion (Pro194Leu)	6	28,494,600	31,806,239	CAPS-C1561-01	CAPS, BciT130 I/Ecc	GCGGCAGTCACGATACCATGAAAGGC	CGACTGGGTCCAGATGATTGGG		13
3	Vegetative organ	Leaf	*Curly leaf2*	*cul-2*	*CsPHB*	*Haplotype2*	Class III homeodomain-leucine zipper (HD-ZIP III) t	Y	EMS	*Csa6G525430*	CsGy6G036200.1	CCMC^{C1134}	SNP (G to A), results in an amino acid substituion (Gly191Glu)	6	28,494,600	31,806,239	dCAPS-C1134-01	dCAPS, DdeI	GCGGCAGTCACGATACCA	CGACTGGGTCCAGATGACTG		13
4	Vegetative organ	Leaf	*Littleleaf*	*ll*	*CsSAP*		Ortholog of Arabidopsis STERILE APETALA (SAP)	Y	Natural	Csa6G111910	CsGy6G009260.1	H19	SNP (T to G), results in an amino acid substitution (Trp264Gly)	6	7,662,821	7,716,895	ll-dCAPS2	dCAPs, DdeI	TACCTGGTCGTGCATTTCACTTA	CCAACTTGCTCCGTTAGCTC		16
5	Vegetative organ	Leaf	*Littleleaf -2*	*ll-2*	n.a		n.a	N, 1.24Mb	Natural	n.a	n.a	XF-24 (*Cucumis sativus*	n.a	7	0.32Mb to 1.55Mb	1,705,258	InDel-4	Indel	AATTTCTTATTCAACAAACCCG	TGTCGTTGATCTATGTGCTCTC		115
6	Vegetative organ	Leaf	*Paternal sorting of mitochondria*	*Psm*	*CsPPR336*		Pentatricopeptide repeat (PPR) protein	Y	Natural	Csa3G824840	CsGy3G036250.1	*Cucumis sativus* var. *ha*	Two SNPs, results in amino acid substitution (P168S and S220R)	3	32,575,257	34,459,723	BsaWI	CAPs, BsaWI	CAGAACTCCAAGACGCCTGT	GCC CTTTGAGCCTCTTCTTT		10,116
7	Vegetative organ	Leaf	*Round leaf1*	*rl-1*	*CsPID*	*Haplotype1*	Serine/threonine kinase	Y	EMS	*Csa1G537400*	CsGy1G024130.1	CCMC^{C356}	SNP (C to T), results in an amino acid substitution (Pro334Ser).	1	19,389,180	22,923,411	rl-1SNP	dCAPs, HhaI	GTCAACGGTTTCTTTATAATGCT	GTACTCACGATACGTCGCG		11
8	Vegetative organ	Leaf	*Round leaf2*	*rl-2*	*CsPID*	*Haplotype2*	Serine/threonine kinase	Y	EMS	*Csa1G537400*	CsGy1G024130.1	CCMC^{C949}	SNP (G to A), results in an amino acid substitution (Glu335Lys)	1	19,389,177	22,923,411	rl-2SNP	dCAPs, Hpy188I	CTGATCCTTAGTTTTCGCCAC	ACTCACGAGTACGTCTCGTCG		11
9	Vegetative organ	Leaf	*Round leaf*	*rl*	*CsPID*	*Haplotype2*	Serine/threonine kinase	Y	EMS	*Csa1G537400*	CsGy1G024130.1	CCMC^{rl}	SNP (G to A), results in an amino acid substitution (Val345Met)	1	19,389,144	22,923,411	n.a	n.a	n.a	n.a		12
10	Vegetative organ	Leaf	*Small and cordate leaf*	*scl-1*	*CsSCL1*		Nucleoside bis-phosphate phosphatase	Y	EMS	*Csa7G062760*	CsGy7G005090.1	406^{scl1}	SNP (G to A), results in an amino acid substitution (Ser to Asn)	7	3,596,452	3,797,553	SNP7G3596452	KASPAR	ACTCACCCGTTAGAGAATATAGTAC	AAACTCACCCGTTAGAGAATATAGTAT	GGCAGCTCGGAGATTTGGTATCAAA	117
11	Vegetative organ	Leaf	*variegated leaf*	*vl*	n.a		n.a	N	EMS	n.a	n.a	Unknown	n.a	6	18.28Mb to 18.67Mb	n.a	n.a	n.a	n.a	n.a		8
12	Vegetative organ	Leaf	*Virescent leaf 1*	*v-1*	*CsCNGCs*		Cyclic-nucleotide-gated ion channel protein	Y	Natural	Csa6G141420	CsGy6G011700.1	9110Gt	SNP (A to T) at promoter region.	6	10,094,568	10,205,338	v1SSR8	SSR	GGCAATGGTAAAGGTTGGAA	AAGGGATGTGTGGTGTGGTT		7
13	Vegetative organ	Leaf	*Virescent yellow leaf*	*vyl*	*CsVYL*		DnaJ-like zinc finger protein	Y	EMS	*Csa4G637110*	CsGy4G021760.1	CCMC^{vyl}	SNP (C to T). Results in an amino acid substitution (Glu73Lys)	4	20,695,774	28,387,102	na	na	na	na		9
14	Vegetative organ	Leaf	*Yellow plant*	*yp*	*CsChlI*	*golden leaf*	CHLI subunit of Mg-chelatase	Y	EMS	*Csa6G518380*	CsGy6G034680.1	CCMC^{C528}	SNP (G to A) in its third exon resulted in an amino acid substitution (G269R)	6	27,511,194	30,831,621	ypSNPC-T	dCAPs, BslI	GAGTAACTGTGGCCTAAGCTCCC	TTTGGATGATCATTTAGTTGACG		6
15	Vegetative organ	Tendril	*Tendril-less*	*ten*	*CsTEN*		TCP (TB1, CYC, and PCF) transcription factor	Y	Natural	*Csa5G644520*	CsGy5G029420.1	CG9192	SNP (A to T), results in an amino acid aubstitution (N338Y)	5	27,045,330	32,763,879	rSNP1-dCAPs	dCAPs,	TTGGAAGCTGGAAAATAGAAAATAT	AATAATATCATTGGGATCATGTGG		14
16	Vegetative organ	Tendril	*tendrilles-1*	*td-1*	*CsGCN5*		Histone acetyltransferase	Y	EMS	*Csa6G527060*	CsGy6G036850.1	CCMC^{8007}	SNP (G to A), results in an amino acid substitution (Asp82Asn)	6	28,883,361	32,202,841	NWdCAPS353	dCAPS, AatII	GGCATCGTCAGAATCGACGT	TACCCACTCGGCTCACCTCA		15
17	Vegetative organ	Architecture	*compact*	*cp*	*CsCullin1*		Cullin-1 protein	Y	Natural	Csa6G197230	CsGy6G014690.1	High wire	SNP (A to G), results in an amino acid substitution (Ade147Gua)	6	12,675,251	12,880,366	n.a	n.a	n.a	n.a		17
18	Vegetative organ	Architecture	*compact1*	*cp-1*	n.a		n.a	N, 178kb	Natural	n.a	n.a	PI308915 (WI7201)	n.a	4	22.17Mb to 22.36Mb	29,878,253	CKX-indel	Indel	GAAACGGTTCCAGGTAAGCA	TCCCATTTCTTTATTTACTTCAGC		18,118
19	Vegetative organ	Architecture	*short internode*	*si*	*CsVFB1*		an F-box protein from VIER F-BOX PROTEIN (VFB)	Y	EMS	*Csa4G641640*	CsGy4G022710.1	406^{si}	SNP (C to T), results in a premature stop codon	4	21,398,058	29,079,919	SNP4G21398058	dCAPs, DraI	TCACTCCGATGAGAACAAGTTTTT	ACGATGCAAACTCTTGAGGAAAC		19
20	Vegetative organ	Architecture	*Super compact1*	*scp-1*	*CsCYP85A1*		BR-C6-oxidase, BR biosynthesis pathway	Y	EMS	Csa5G641590	CsGy5G028960.1	CCMC^{C257}	SNP (G to A), results in a tryptophan codon at 157 position (TGG) substitution to a	5	26,718,684	32,438,112	P450-dCAPS	dCAPs, DdeI	ATGCGATCCCATTTGAGTACTT	TGCCGAGGACTAACTTAAAGAAC		20
21	Vegetative organ	Architecture	*Super compact2*	*scp-2*	*CsDET2*		Steroid 5-alpha-reductase	Y	Natural	Csa3G732550	CsGy3G029480.1	AM204M	SNP (C to T), results in amino acid substitution (R182W)	3	27,762,276	29,959,460	S433755	dCAPs,	ACGAACAAACCTGTCGGGAT	CTACATTGTTGCGGGGGAGA		21
22	Vegetative organ	Trichome	*Glabrous1*	*gl1*	*CsGL1*	*syn.Mict*	Homeodomain-leucine zipper I (HD-Zip I) protein	Y	Natural	*Csa3G748220*	CsGy3G031820.1	Daqingba^{gl1}	Deletion. A 2649 bp fragment deletion from -189 to 2460 bp of the start codon of	3	29,041,903	31,245,240	Gl1g	Indel	TTTGTGCCACATGAACCC	TCTGAACTGTCCAAACGG		24
23	Vegetative organ	Trichome	*Micro-trichome*	*mict*	*CsGL1*	*syn.gl1*	Homeodomain-leucine zipper I (HD-Zip I) protein	Y	Natural	*Csa3G748220*	CsGy3G031820.1	North China inbred line	Deletion. A 2649 bp fragment deletion from -189 to 2460 bp of the start codon of	3	29,041,903	31,245,240	Gl1g	Indel	TTTGTGCCACATGAACCC	TCTGAACTGTCCAAACGG		25
24	Vegetative organ	Trichome	*Glabrous2*	*gl2*	n.a		n.a	N, 0.6cM	Natural	*n.a*	na	NCG042	n.a	2	14.67Mb	20,772,692	SSR10522	SSR	TTCCTTTTGTTTTTGGTATGGG	ATGTCTGCTTTGCTGGCTTT		26
25	Vegetative organ	Trichome	*Glabrous3*	*gl3*	*CsGL3*	*Variant2*	Homeodomain-leucine zipper IV (HD-Zip IV) prote	Y	Natural	*Csa6G514870*	CsGy6G033240.1	NCG157	SNP(T611C, G820A, G865A) at 4th exon.	6	26,734,859	30,057,625	InDel-19	Indel	CATTTGACCAAACGCACACT	TGTCACAGCACAATGGACCT		28
26	Vegetative organ	Trichome	*Glabrous3*	*gl3*	*CsGL3*	*Variant3*	Homeodomain-leucine zipper IV (HD-Zip IV) prote	Y	Natural	*Csa6G514870*	CsGy6G033240.1	WI7412	5-kb long terminal repeats (LTR) retrotransposon in 4th exon.	6	26,734,859	30,057,625	GL3_2	Indel	TGGGCATCACTAACTTCGGT	ACAGTGGACCATTGCGTCTA		27
27	Vegetative organ	Trichome	*Trichome-less*	*tril*	*CsGL3*	*Variant4*	Homeodomain-leucine zipper IV (HD-Zip IV) prote	Y	Natural	*Csa6G514870*	CsGy6G033240.1	CGN19839 (Teplicnyi At	Insertion in 2nd exon caused gene function lost.	6	26,734,859	30,057,625	P1	Indel	TCGGACACAAAACCTTACAAATG	AGTCAATGCCCGAACCTCCT		29
28	Vegetative organ	Trichome	*glabrous4*	*gl4*	*CsGL4*	*Tsp; Variant1*	C-type lectin receptor-like kinase protein	Y	EMS	Csa1G056960	CsGy1G010080.1	406^{gl2}	SNP (G to A), results in an amino acid substitution (Gly212Arg)	1	6.00Mb to 6.72Mb	6,247,822	APS-SNP7	dCAPs, BSeNI	AGTATCTAGTTATTCTAGCTGTTGTGACT	CAGAAGCTGCAGGGTTGGAT		2
29	Flower	Flower structure	*Extra long sepal*	*els*	*CsSEP2*		Homolog of Arabidopsis SEPALLATA2	Y	EMS	Csa4G126690	CsGy4G009560.1	406^{els}	SNP (C to T) at the splicing site, results in the deletion of exon 6 (42bp)	4	7,741,482	7,996,718	SNP4G7741482	dCAPs	AACTAACAGAGCTCTGCAAATGAAG	TTCTTAAAGTTTGAGAATCAGTAAATT		5
30	Flower	Flowering time	*Early flowering time*	*qef1.1*	*CsFT*		Homolog of the Arabidopsis thaliana FT	Y	Natural	Csa1G651710	CsGy1G030960.1	Muromskij	*	1	25,850,971	29,537,849	na	na	na	na		43
31	Flower	Male sterility	*Male sterility*	*ms-3*	*ms-3*		Homeodomain (PHD) finger protein	Y	Natural	Csa3G006660	CsGy3G001080.1	YL-5	SNP (T to G), results in an amino acid substitution (Tyr420Asp)	3	806,246	804,179	A806246C	KASPAR	GCCATTTCTAAGTTCATGAATC GTGTA	CCATTTCTAAGTTCATGAATCG TGTC	TAGTTCGCCGTGCA GTGAATCCTAA	119
32	Flower	Sex expression	*Androecious*	*A*	*CsACS11*		1-aminocyclopropane-1-carboxylic acid synthase	Y	Natural	Csa2G353460	CsGy2G018140.1	Erez	1–bp deletion leads to a premature stop codon	2	16,345,626	27,954,918	A81/A85	SSR	GATGGAGCCAACATGAATGCTCAG	AAGAAGTTATTGGGATCGAGCAAG		36,120
33	Flower	Sex expression	*Androecious-1*	*a-1*	*CsACO2*		1-aminocyclopropane-1-carboxylate (ACC) oxidas	Y	EMS	Csa6G511860	CsGy6G032740.1	406^{a}	SNP (C to T), results in an amino acid substitution (P255S)	6	26,464,719	29,787,615	SNP6G26464719	dCAPs, EcoRI	AGAATGTCGATAGCATCATTCT AGAAT	CTTGGCTCTTTTGCTTGAAACTT		38
34	Flower	Sex expression	*Andromonoecious*	*m*	*CsACS2*	*Haplotype1*	1-aminocyclopropane-1-carboxylic acid synthase	Y	Natural	Csa1G580750	CsGy1G027100.1	H34	SNP (G to T), results in an amino acid substitution (G33C)	1	21,855,870	25,520,318	S_ME8SA7	SCAR	CGATAACAAGCCGCTGAGGAGAT	GTGGCCCGTTACCACTAATAC		35
35	Flower	Sex expression	*Andromonoecious*	*m-1*	*CsACS2*	*variant of m locus*	1-aminocyclopropane-1-carboxylic acid synthase	Y	Natural	Csa1G580750	CsGy1G027100.2	H38	A 14 bp deletion, results in premature stop codon	1	21,938,479	25,520,318	CsACS2_CDS	SCAR	CTCAAGGAAAAAAGAACAAACATCAA	CGTCAGATGGATTGTTAGGTGGTTA		64
36	Flower	Sex expression	*Femaleness*	*F*	*CsACS1G*		1-aminocyclopropane-1-carboxylic acid synthase	Y	Natural	Csa6G496970	CsGy6G028780.1		duplications	6	24,245,649	27,588,957	CsACS1.2s	SCAR	TTAACTACTTCGGACGGGCATAG	AGGTGTTCAGCAAACATAGGGTG		37
37	Flower	Sex expression	*sub-gynoecious*	*sgy*	n.a	*sgy3.1*	n.a	N, 799kb	Natural	n.a	na	S-2-98	n.a	3	12.16Mb to 12.95Mb	12,186,834	C3D2	dCAPs, HpaII	CGTATTTCTTCTTCTTCGTCAAACATTCCG	TTTGTAGTGAAGAAGGAGAAGGAGATGGGG		39
38	Fruit	Peduncle direction	*upward-pedicel*	*up*	*CsUP*		Auxilin-like protein with a conserved DnaJ-domair	Y	Natural	Csa1G535800	CsGy1G024010.1	C-8-6^{up}	5-bp deletion, results in a frameshift mutation and a premature stop codon	1	19,204,081	22,736,083	Indel-CsUp	Indel	AACCAATGTCCCAACACAATG	GGGAGAGATGAGGTGCAAACT		121
39	Fruit	Size and shape	*Carpel number*	*cn*	*CsCLV3*		CLAVATA3	Y	Natural	Csa007092	CsGy1G014910.1	TrueLemon	multiple SNPs within CsCLV3	1	10,551,968	10,816,556	CN_D1	dCAPs, BccI	ATGGACGTCTACAAAACATGGCTACCCAT	TCCAGCACTCATCACCAACT		65
40	Fruit	Size and shape	*Fruit size1.2*	*qfs1.2*	*CsSUN2*		Tomato fruit shape gene SUN homolog	Y	Natural	Csa1G575000	CsGy1G026840.1	WI7239	161-bp deletion, results in a truncated protein	1	21,748,440	25,331,715	Sun_indel	Indel	GAAATCCTTCTACTCCGAAACAGA	TTCCTCGCCTAATTTCACCAA		68
41	Fruit	Size and shape	*Fruit size2.1*	*qfs2.1*	*CsTRM4*		Ortholog of AtTRM5/SlTRM5 (TON1 RECRUIT MO	Y	Natural	Csa2G227860	CsGy2G011350.1	WI7239		2	11,042,204	11,224,424	Xl2snp10*	dCAPs, TaqI	ATGATCAATTCCTTATTAGATTAT	TCAAGTGCAACTTATTCCAAGC		69
42	Fruit	Size and shape	*Fruitfull*	*ful*	*CsFUL1*		MADS-box	Y	Natural	Csa1G039910	CsGy1G006040.1	Many Cultivars	A SNP (C3393A), results in an amino acid substitution(Lys168Glu)	1	3,960,104	3,947,246	na	na	na	na		62
43	Fruit	Size and shape	*Mango fruit*	*mf*	*CsWOX1*		WUSCHEL-related homeobox1 (WOX1)	Y	Natural	Csa1G042780	CsGy1G007020.1	AM218(Extra Early Maje	1-bp deletion leads to a premature stop codon, result in a truncated protein with 1	1	4,494,646	4,488,841	UW026993	SSR	CTCATGTCTCCCTACAGACTCG	TCTCGACATAGATGCTAATACCAA		66
44	Fruit	Size and shape	*Short fruit-1*	*sf-1*	n.a		n.a	N, 174.3kb	Natural	n.a	n.a	CNS2^{sf1}	n.a	6	11.58Mb to 11.76Mb	11,696,118	STS1	sequence-tagged sit	TGTTTGGCCCAAGGAGTT	GTAACTTGCATGCGAGGT		63
45	Fruit	Size and shape	*Short fruit -2*	*sf-2*	*CsSF2*		RING-type E3 ligase	Y	EMS	Csa2G174140	CsGy2G010390.1	406^{sf1}	A SNP (G to A), resustls in an amino acid substitution (Arg230Lys).	2	9,983,495	10,113,710	Chr2-9983495-dcaps	dCAPs	CCTCGTCTAGTTATTCCATTGT	TCCCAGGAGGTGTGGCAACTCCGTCTTAGC		4
46	Fruit	Fruit flesh thickness	*Fruit flesh thickness2.1*	*qfth2.1*	*fth2.1*		Protein-lysine methyltransferase (PKMT)	N	Natural	n.a	n.a	XUE1, Pepino, Jin5-508,	n.a	2	4.41Mb to 4.60Mb	4,434,893	na	na	na	na		72
47	Fruit	Flesh color	*Orange flesh*	*ore*	*CsBCH1*		β-carotene hydroxylase	Y	Natural	Csa3G183920	CsGy3G017310.1	CG9164	SNP (), results in an amino acid substitution (Ala257Asp)	3	12,845,300	13,300,278	SNP3G12845300	na	na	na		33
48	Fruit	Flesh color	*Yellow flesh*	*yf*	n.a		n.a	N, 149kb	Natural	n.a	n.a	PI200815	n.a	7	15.13Mb to 15.28Mb	19,537,576	yfSSR108	SSR	TTTGAGGGCACTCACAAGC	CATTCGATCGATGGTGGATT		54
49	Fruit	Epidermal feature	*Black spine*	*B*	*CsMYB60*	*Pleiotrphy of B*	R2R3-MYB transcription factor	Y	Natural	Csa4G003090	CsGy4G001040.1	WI7200	1-bp insertion (T) in the intron.	4	620,189	635,650	MYB-1dCAPS	dCAPs	GGAAACAGGTAATTTATTGGACA	TATTTTAACATGTGTAATTTAAAATA		52,53
50	Fruit	Epidermal feature	*Few spines*	*fs1*	*CsGL3*	*Variant1*	Homeodomain-leucine zipper IV (HD-Zip IV) transc	Y	Natural	*Csa6G514870*	CsGy6G033240.1	CNS2^{CNS5}	Insertion. A 812 bp Insertion in the *CsGL3* promoter in CNS5.	6	26,734,859	30,057,625	MM3	Indel	ATTGAATGTGGGTCTGGC	GTTGTTGTTGGTCAGTTCCA		56
51	Fruit	Epidermal feature	*Numerous spines*	*ns*	*CsLAX3*		Auxin transporter-like protein 3	Y	Natural	Csa2G264590	CsGy2G013240.1	NCG122	SNP (A to G), results in an amino acid subsititution (V to I); 1-bp deletion, results ir	2	12,795,994	13,010,523	nsIndel54	Indel	GGCTGTTCCTATCAATTAACC	TGTTTGCTTCCCAAGTTCA		58,59
52	Fruit	Epidermal feature	*tender spines*	*tsp*	*CsTsp*	*gl4; Variant2*	C-type lectin receptor-like kinase protein	Y	Natural	Csa1G056960	CsGy1G010080.1	NC073	SNP (GT to GC) at alternative slicing, results in a N-terminal deletion.	1	6,245,947	6,251,039	na	na	na	na		3
53	Fruit	Epidermal texture	*Heavy netting*	*H*	n.a		n.a	N, 1.2Mb	Natural	n.a	n.a	9110Gt	n.a	5	19.93Mb to 20.45Mb	25,709,527	n.a	n.a	n.a	n.a		122,123
54	Fruit	Epidermal texture	*Palisade epidermis*	*Pe*	n.a		n.a	N, 227.5kb	Natural	n.a	n.a	NCG121	n.a	5	20.03Mb to 20.26Mb	25,915,175	SSR14611	SSR	ATTTGGCTTCCAGGTTGATG	GAATCGAAAACCGATGAGGA		124
55	Fruit	Epidermal feature	*Light green fruit*	*lgf*	*CsYcf54*		Ycf54-like protein	Y	EMS	Csa6G133820	CsGy6G010900.1	406^{lgf}	SNP (C to T), results in an amino acid substitution (Pro203Ser).	6	9,285,631	9,382,904	SNP6G9285631	dCAPs, HindIII	GAAGCCTGAAAATTGGGAAGCT	ACGAAATCCTCACACCATCAA		51
56	Fruit	Epidermal feature	*Light green peel*	*lgp*	*CsARC5*		ACCUMULATION AND REPLICATION OF CHLOROP	Y	EMS	Csa7G051430	CsGy7G004650.1	406^{lgp}	SNP (C to T), results in an amino acid substitution (Arg213Lys)	7	3,286,953	3,474,053	SNP7G3286953	dCAPs, StuI	AGTTTGGTTGATACAATCACAGGC	TGACTTTGTCATCAGTTCTTTATTT		50
57	Fruit	Epidermal feature	*Red fruit color*	*allelic to B*	*allelic to B*			Y	Natural													52
58	Fruit	Epidermal feature	*Uniform immature fruit color*	*u*	n.a		n.a	N, 313.2kb	Natural	n.a	n.a	S94, S98	n.a	5	20.01Mb to 20.32Mb	25,663,570	SSR27	SSR	TATGAATAAAGGATGAGGTG	ATTGTGACATTGTTAGCCA		125
59	Fruit	Epidermal feature	*White skin color*	*w*	*CsAPRR2*		ARABIDOPSIS PSEUDO RESPONSE REGULATOR2-L	Y	Natural	Csa3G904140	CsGy3G044470.1	Q24	1-bp insertion (G) resulted in a premature stop codon that is hypothesized to have	3	39,257,726	41,175,650	pan3925	InDel	AATTGTATTTATTTGTAGCACAT	GAGTAATACATTTCAACCACAC		49,126
60	Fruit	Epidermal feature	*Yellow green peel*	*ygp*	*CsMYB36*		MYB transcription factor	N	EMS	Csa2G352940	n.a	ygp	SNP (C to T), results in an amino acid substitution (Leu to Phe)	2	16,323,852	27,932,225	SNP16324218	KASPAR	CTGTAGGTTGAGATGGTTGAATTATC	GCTGTAGGTTGAGATGGTTGAATTATT	CTTCTTCAGAGAAACCTCCATGCTTAAT	127
61	Fruit	Epidermal feature	*Tubercle size*	*Ts1*	*CsTs1*		Oleosin protein	Y	Natural	Csa5G523090	CsGy5G017890.1	DRT468	Sequence change in the promoter of CsTS1 gene.	5	18,474,911	24,119,025	YS-1	KASPAR	GAAGGTGACCAAGTTCATGCTTTTTTTTTATC	GAAGGTCGGAGTCAACGGATTTTTTTTTATC	GAAGTAAAGATGACACGAAAAAAGTCCAATAG	61
62	Fruit	Epidermal feature	*Warty*	*Tu*	*CsTu*		Transcription factor with a single C2H2 zinc finger	Y	Natural	Csa016861	CsGy5G019590.1	S52	This gene is absent in non-warty line S06.	5	20,292,161	25,945,387	C3	Indel	TGTACGAATAGCTACCTCCAAAAGAAATG	CCACCATTTTGGCACATTTTTTCTC		60
63	Fruit	Epidermal texture	*Dull fruit skin*	*D*	n.a		n.a	N, 244.9Kb	Natural	n.a	n.a	S94, S52	n.a	5	20.54Mb to 20.79Mb to	26,438,292	SSR37	SSR	TCCTCATCATTGCCACTC	CTTCAACGCCTGATTCTG		122,128
64	Abiotic stress tolerance	Waterlogging	*Adventitious root number (ARN)*	*qARN6.1*	*CsARN6.1*		AAA ATPase domain-containing protein	Y	Natural	Csa6G504460	CsGy6G030800.1	Zaoer-N	SNP (A to G), results in an amino acid substitution (Asp506Gly)	6	25,496,855	28,825,007	SNP02	dCAPs, TaqI	GCGGTTCTTGGGGGACTGGTCG	CTATTTCTCAATTCCCAGTTT		111,112
65	Disease resistance	Bacterial resistance	*Resistance to Pseudomonas syringae pv. Lachrymans (ALS)*	*psl*	*CsSGR*	allelic to dm1	Stay-green (Mg dechelatase)	Y	Natural	Csa5G156180	CsGy5G003280.1	Gy14/WI2757	SNP(A to G), results in an amino acid substitution (Glu108Arg)	5	5,462,918	2,149,251	SNP08	dCAPs, BsrBI	TTTTCTGGAATGGGTGTTAAA	cattctcCTACCACTTCATCCGCT		79
66	Disease resistance	Fungal resistance	*Powdery mildew resistance (PM)*	*pm1.1*	n.a		n.a	N, 41.1kb	Natural	n.a		Jin5-508	n.a	1	6.81Mb to 6.86Mb	6,841,559	SNP09	dCAPs, DdeI	GACCATACAAATAACAGAATTCTAA	TTGCTTTTGTTTTCTTGTAC		86
67	Disease resistance	Fungal resistance	*Powdery mildew resistance (PM)*	*pm-s*	n.a		n.a	N, 135.7kb	Natural	n.a		NCG-122/WI2757	n.a	5	24.71Mb to 24.84Mb	30,406,396	pmSSR27	SSR	CCCTATGGTGAAAGCCACT	CGCCTCAAACCCATACCCAA		129
68	Disease resistance	Fungal resistance	*Powdery mildew resistance (PM)*	*pm5.3*	n.a		n.a	N, 468.0kb	Natural	n.a		IL52 (*C. Hystrix*)	n.a	5	24.55Mb to 25.02Mb	30,434,472	SNP6	dCAPs,	TTTCTTGTACAGGGGAAGGTGG	TGTTTTGGTCAGGCAAAGGGT	TGCCTCTCTTCTTCTCTCTTTTGCAT/CTCTTTTTTCC	85
69	Disease resistance	Fungal resistance	*Powdery mildew resistance (PM)*	*pm*	*CsMLO1*	*Haplotype1*	Cell membrane protein of mildew locus O (MLO)	Y	Natural	Csa5G623470	CsGy5G026660.1	S1003	A 1449-bp insertion results in a loss of gene function	5	24,831,373	30,524,541	N7F-N14R	SCAR	TTCATTTCCTCTCCTTATTGGT	CATCGTTTTGTTTTAGATTGGAA		82–84
70	Disease resistance	Fungal resistance	*Powdery mildew resistance (PM)*	*pm*	*CsMLO1*	*Haplotype2*	Cell membrane protein of mildew locus O (MLO)	Y	Natural	Csa5G623470	CsGy5G026660.1	R077	SNP (GT to GC) at alternative slicing, results in truncated transcript without exon 5	5	24,831,373	30,524,541	na	na	na	na		82,83
71	Disease resistance	Fungal resistance	*Powdery mildew resistance (PM)*	*pm*	*CsMLO1*	*Haplotype3*	Cell membrane protein of mildew locus O (MLO)	Y	Natural	Csa5G623470	CsGy5G026660.1	PI 197088	1-bp insertion (A), results frame shift (FS488) and premature stop codon	5	24,831,373	30,524,541	na	na	na	na		82,83
72	Disease resistance	Fungal resistance	*Resistance to Cladosporium cucumerinum (Scab)*	*ccu*	n.a		n.a	N. 180kb	Natural	n.a		9110Gt	n.a	2	2.59Mb to 2.79Mb	3,276,171	SSR17631	SSR	TTGATTCCAATTTCATTACTTTTCA	TTCCCTAAGTAGTGACGGATTTTT		90,122
73	Disease resistance	Fungal resistance	*Resistance to Colletotrichum lagenarium (Anthracnose)*	*cla*	*CsSGR*	allelic to dm1	Stay-green (Mg dechelatase)	Y	Natural	Csa5G156180	CsGy5G003280.1	Gy14/WI2757	SNP(A to G), results in an amino acid substitution (Glu108Arg)	5	5,462,918	2,149,251	SNP08	dCAPs, BsrBI	TTTTCTGGAATGGGTGTTAAA	cattctcCTACCACTTCATCCGCT		78,79
74	Disease resistance	Fungal resistance	*Resistance to Corynespora cassiicola* (TLS)	*cca-2*	n.a		n.a	N, 1.25Mb	Natural	n.a		PI183967(*Cucumis sativ*	n.a	6	9.37Mb to 10.65Mb	9,468,049	SSR21318	SSR	GACACCCCATTCCTCATCAT	GCTTCATCACTCCCAATTCAG		130
75	Disease resistance	Fungal resistance	*Resistance to Corynespora cassiicola* (TLS)	*cca-3*	*cca-3*		CC-NB-ARC type protein	Y	Natural	Csa6G375730	CsGy6G019440.1	D31	SNP(C to T), results in an amino acid substitution (His440Tyr)	6	16,936,147	19,877,323	6M375730_NonsynSNP1	KASPAR	TGCATTATTCTCCAAAGATTATCACC	GTGCATTATTCTCCAAAGATTATCACT	CCTTGTGCCATCCATTGTTTTATCAAATCA	88
76	Disease resistance	Fungal resistance	*Resistance to Corynespora cassiicola* (TLS)	*cca-1*	n.a		n.a	N, 2.9cM	Natural	n.a		Q5	n.a	6	15.11Mb	17,894,751	CSFR33	EST-SSR	GAGCATTGAGCAGTCACGAG	GTATTTGTGAGGGGTGGAGG		87
77	Disease resistance	Fungal resistance	*Resistance to Fusarium oxysporum f. sp. Cucumerinum (Fusa*	*Foc*	n.a		n.a	N, 740Kb	Natural	n.a		9110Gt	n.a	2	2.52Mb to 3.26Mb	3,276,171	SSR17631	SSR	TTGATTCCAATTTCATTACTTTTCA	TTCCCTAAGTAGTGACGGATTTTT		89
78	Disease resistance	Oomycte resistance	*Resistance to Pseudoperonospora cubensis (DM)*	*dm*	*CsSGR*		Stay-green (Mg dechelatase)	Y	Natural	Csa5G156180	CsGy5G003280.1	Gy14/WI2757	SNP(A to G), results in an amino acid substitution (Glu108Arg)	5	5,462,918	2,149,251	SNP08	dCAPs, BsrBI	TTTTCTGGAATGGGTGTTAAA	cattctcCTACCACTTCATCCGCT		79
79	Disease resistance	Oomycte resistance	*Resistance to Pseudoperonospora cubensis (DM)*	*dm4.1*	n.a		n.a	N	Natural	n.a		WI7120	n.a	4	15.42Mb to 15.74Mb	22,679,946	CsDM4-066	SSR	GAGGGAGTTGGTCACTCCAA	AAAAGGCATTATGAGCCTGA		80
80	Disease resistance	Oomycte resistance	*Resistance to Pseudoperonospora cubensis (DM)*	*dm5.2*	n.a		n.a	N	Natural	n.a		WI7120	n.a	5	16.26Mb to 16.93Mb	23,380,844	CsDM4-055	SSR	TTGTCCTTCACTTTCAACTTTTACA	TCGATGTATGATTGTGGCACT		81
81	Disease resistance	Virus resistance	*Resistance to cucumber mosaic virus (CMV)*	*cmv6.1*	n.a		n.a	N, 1.62Mb	Natural	n.a		02245	n.a	6	8.00Mb to 9.03Mb	7,688,887	SSR9-56	SSR	CAAAACAAGGTAAGGTGTATTGGA	TAAAAGGCAGGACATGCTCA		95
82	Disease resistance	Virus resistance	*Resistance to Papaya ringspot virus (PRSV)*	*PRSV*	n.a	known as watermelon	n.a	N, 1.8cM	Natural	n.a		02245	n.a	6	9.03Mb to 9.63Mb	9,726,336	SSR11-177	SSR	CGTGGCATAAAACCACGAAT	CAAATTCAACAAAACCCTACCA		93
83	Disease resistance	Virus resistance	*Resistance to watermelon mosaic virus (WMV)*	*wmv*	n.a		n.a	N, 134.7kb	Natural	n.a		02245	n.a	6	19.36Mb to 19.49Mb	22,530,869	SSRWMV60-23	SSR	TGCGTATGCCCTTGTATCCT	AAAAGGGACTGGTGTAGACTGC		93
84	Disease resistance	Virus resistance	*Resistance to Zucchini Yellow Mosaic Virus (ZYMV)*	*zym*	*CsVPS4*	*zym*$^{A192-18}$, *zym*^{Dina}, *z*	Vacuolar protein sorting-associated protein 4 (VP!	Y	Natural	Csa6G152960	CsGy6G012710.1	A192-18, Dina, TMG-1	Two SNP (T86C and G99A), results in amino acid substitution (Phe29Ser and Met3	6	10,850,939	10,962,805	CAPS-T86C	CAPs, MnlI	AGCAGGCGGTACATGAAGAT	CCTGTTCTGGATCCTCTCCA		91,92
85	MISC	Bitterness	*bitter fruit*	*bt*	*CsBt*		Basic helix-loop-helix (bHLH) transcription factor	Y	Natural	Csa5G157230	CsGy5G003340.1	PI 183967	SNP (G to A) and a 699-bp insertion at promoter region	5	5,513,390	2,198,903	na	na	na	na		113
86	MISC	Bitterness	*bitter leaf*	*bl*	*CsBl*		Basic helix-loop-helix (bHLH) transcription factor	Y	Natural	Csa5G156220	CsGy5G003320.1	XY-3; E3-231	SNP (G to C) at splicing site of the predicted intron that likely disrupts proper gene	5	5489366	2,175,005	na	na	na	na		113
87	MISC	Bitterness	*bitterfree*	*bi*	*CsBi*		Cucurbitadienol synthase	Y	Natural	Csa6G088690	CsGy6G007190.1	9110Gt; CG7744	SNP (G to A), results in an amino acid substitution(C393Y); 1–base pair (bp) deletic	6	6,088,655	6,147,641	na	na	na	na		113
88	MISC	Fragrance	*Fragrance*	*fgr*	*CsBADH*		Betainealdehyde dehydrogenase 2	Y	Natural	Csa1G006270	CsGy1G001790.1	PK2011T202	SNP (A to G), results in an amino acid substitution (Tyr163Cyt)	1	162,652	1,165,392	CsBADH2-A1	SNAP	GCTGATGTCTATCTTTTATTGAAGGAACAA	CACAATAAATAAAGTTCATAAAATCCATAATCAC		114,131

Table S2. Summary of disease resistance QTL identified in cucumber (as of July 2019)

Consensus QTL	PVE (%)*	Chr	LOD Interval (Gy14 V2.0)**		Source of Resistance	QTL names in reference and notes	References
			Position-Left	Position-Right			
**Resistance to powdery mildew (PM)**							
pm1.1	23.2-49.6%	1	5,876,498	22,503,767	PI 197088	Effective at 20 °C and 26 °C	97
pm1.1	44.0%	1	8,222,187	22,234,860	PI 197088	Effective at 20 °C and 26 °C	96
pm1.1	16.9%	1	2,163,238	17,722,258	WI 2757	*pm-tl1.1, pm-cl1.1,pm1.1*	100
pm1.2	7.0%	1	17,250,996	23,465,958	S06		98
pm1.2	14.6%	1	18,577,265	21,339,881	WI 2757	*pm-tl1.2*	100
pm1.3	24.0%	1	22,234,860	25,397,685	PI 197088	*pm1.2*	96
pm1.3	n/a	1	23,500,000	24,500,000	H136	Detected with BSA	132
pm1.4	3.8%	1	28,477,693	32,220,160	PI 197088	*pm1.1*	81
pm2.1	5.1%	2	8,638,158	13,134,937	PI 197088		81
pm2.2	5.0%	2	14,949,352	15,870,243	PI 197088	*pm2.1*	96
pm3.1	9.0%	3	992,960	5,926,467	PI 197088	*pm3.1*	96
pm3.2	8.5%	3	26,651,364	36,926,708	WI 2757	*pm-hy3.1,*	100
pm4.1	20.8%	4	8,006,703	9,002,834	S06		98
pm4.2	11.3%	4	10,211,019	22,100,020	WI 2757	*pm-hy4.1, pm-cl4.1*	100
pm4.3	11.0%	4	22,517,248	30,262,032	PI 197088	*pm4.1*	96
pm5.1	7.3%	5	11,801,331	20,837,862	K8		99
pm5.1	9.2%	5	11,282,605	16,961,924	S06		98
pm5.1	37.0%	5	16,961,924	20,847,180	PI 197088	*pm5.2;* Effective at 20 °C	96
pm5.2	41.0%	5	21,395,304	24,124,874	WI 2757	*pm-cl5.1, pm-clhy5.1, pm-tl5.1*	100
pm5.3	18.1%	5	27,072,020	31,877,374	PI 197088	LG IV, (*pm-l, pm-s, pm-h*); Effective at 20 °C	97
pm5.3	n/a	5	29,379,472	n/a	WI 2757	Treated as a single gene	133
pm5.3	41.6%	5	27,819,449	29,369,915	K8		99
pm5.3	74.5%	5	27,072,020	30,877,374	WI 2757	*pm-cl5.2, pm-clhy5.2, pm-tl5.2*	100
pm5.3	8.0%	5	27,688,515	33,372,091	PI 197088	*pm5.3;* Effective at 20 °C and 26 °C	96
pm5.3	30.5%	5	30,344,255	31,930,132	PI 197088		81
pm5.3	n/a	5	30,247,005	30,714,150	IL52		85
pm5.4	19.0%	5	399,606	3,704,782	PI 197088	*pm5.1;* Effective at 26 °C	96
pm6.1	24.0%	6	5,309,327	17,894,771	PI 197088		96
pm6.2	n/a	6	21,000,000	22,000,000	H136	Detected with BSA	132
pm6.3	22.8%	6	803,780	21,455,099	PI 197088	LGI; Effective at 26 °C	97
pm6.3	15.3%	6	27,052,131	27,270,372	S06	co-localized with *F* locus;	98
pm6.3	5.2%	6	27,734,744	27,799,589	K8		99
pm6.3	5.5%	6	27,144,746	29,819,946	PI 197088		81
pm7.1	15.6%	7	12,961,521	13,520,827	PI 197088	LGIII, Effective at 20 °C;	97
**Resistance to downy mildew (DM)**							
dm1.1	36.4%	1	2,163,238	10,024,077	S94	*lg1.1*	134
dm1.1	18.6%	1	2,135,801	4,753,038	K8		135
dm1.1	n/a	1	1,093,041	5,876,498	PI 197088		101
dm1.1	6.4%	1	1,205,471	3,115,794	CCMC, IL52		85
dm1.2	n/a	1	8,761,340	18,377,790	PI 197088		101
dm1.2	4.2%	1	8,761,340	16,421,791	CCMC, IL52		85
dm1.3	n/a	1	22,234,860	24,873,748	PI 197088		101
dm1.3	4.9%	1	19,102,866	30,206,683	PI 197088	*dm1.1*	81
dm1.3	10.6%	1	18,617,607	27,582,938	PI 197088	*dm1.1*	102
dm1.3	4.9%	1	23,626,789	25,436,487	CCMC, IL52		85
dm2.1	24.0%	2	4,145,724	6,300,028	PI 197088	*QTL1, dm2.1*	136
dm2.1	7.3%	2	5,846,714	11,865,405	WI7120	*dm2.1*	80
dm2.1	28.1%	2	6,515,685	10,440,388	TH118FLM	Resistance derived from 'Malini'	137
dm2.1	6.3%	2	8,085,485	10,959,944	PI 197088		81
dm2.2	24.0%	2	29,652,065	30,141,478	TH118FLM	Resistance derived from 'Malini'	137
dm2.2	2.0%	2	31,235,691	33,294,188	PI 197088	*dm2.2*	81
dm3.1	n/a	3	1,122,921	5,926,467	PI 197088		101
dm3.2	7.0%	3	8,049,881	11,937,537	PI 197088	*dm3.1*	102
dm3.2	2.7%	3	9,186,866	10,272,645	PI 197088	*dm3.1*	81
dm3.2	n/a	3	10,578,869	14,966,371	PI 197088		101
dm3.3	5.3%	3	38,606,402	41,586,177	PI 197088	*dm3.2*	81

dm4.1	40.6%	4	21,737,195	23,007,082	WI7120	*dm4.1*	80
dm4.1	13.7%	4	21,589,735	23,442,656	PI 197088		81
dm4.1	27.0%	4	22,226,625	28,307,174	PI 197088	*dm4.1*	102
dm4.1	21.0%	4	5,095,210	27,700,438	PI 197088	*QTL2, dm4.1*	136
dm1	35.0%	5	2,148,763	n/a	WI2757	*CsSGR*	79
dm5.1	4.8%	5	11,282,605	14,799,230	S94	*lg6.1*	134
dm5.1	19.7%	5	7,722,007	11,524,398	S94	*lg6.2*	134
dm5.1	n/a	5	399,606	6,866,682	PI 197088		101
dm5.1	11.7%	5	Unknown	3,483,981	PI 197085		138
dm5.1	27.2%	5	6,598,911	19,170,007	TH118FLM	Resistance derived from 'Malini'	137
dm5.1	12.5%	5	2,656,332	11,013,650	PI 197088	*dm5.1*	102
dm5.1	19.7%	5	7,722,007	11,524,398	PI 197088		81
dm5.1	32.9%	5	920,109	3,291,619	CCMC, IL52		85,139
dm5.2	27.8%	5	17,923,802	23,783,970	PI 197088	*QTL4, dm5.1*	136
dm5.2	19.6%	5	20,837,862	27,819,449	K8		135
dm5.2	n/a	5	22,362,864	26,221,218	PI 197088		101
dm5.2	12.8%	5	Unknown	27,819,449	PI 197085		138
dm5.2	20.8%	5	17,923,802	23,599,240	WI7120	*dm5.1*	80
dm5.2	27.8%	5	22,016,973	23,783,970	PI 197088		81
dm5.2	39.2%	5	21,649,393	23,017,114	WI2757	*Necrosis resistance*	79
dm5.3	n/a	5	27,819,449	33,372,091	PI 197088		101
dm5.3	13.2%	5	31,258,717	33,209,500	PI 197085		138
dm5.3	7.6%	5	28,001,113	28,725,975	PI 197088	*dm5.2*	102
dm5.3	31.0%	5	30,603,948	31,930,132	PI 197088		81
dm5.3	31.3%	5	28,328,355	31,451,421	CCMC, IL52		85,139
dm6.1	5.3%	6	3,725,502	5,919,221	PI 197088		81
dm6.2	n/a	6	9,340,981	10,801,545	PI 197088	*dm6.1*	101
dm6.3	4.5%	6	15,864,566	24,100,408	WI7120	*dm6.1*	80
dm6.4	7.6%	6	30,502,961	31,132,697	K8		135
dm6.4	3.7%	6	29,280,558	31,406,890	WI7120	*dm6.2*	80
dm6.4	3.3%	6	27,501,170	31,289,674	PI 197088	*dm6.2*	81
dm6.4	3.7%	6	26,616,358	29,525,708	CCMC, IL52	*dm6.1*	85,139
dm7.1	n/a	7	21,960,482	23,752,082	PI 197088		101
**Resistance to Angular Leaf Spot (ALS)**							
als1.1	15.4%	1	16,875,551	21,339,881	WI2757	*psl1.1*	79
als3.1	7.6%	3	187,602	21,276,896	WI2757	*psl3.1*	79
psl	28.1%	5	2,148,763	n/a	WI2757	*CsSGR*	79,140
**Resistance to Fusarium Wilt (FOC)**							
Foc2.1	64.2%	2	1,718,098	3,296,126	9110Gt		89
Foc3.1	n/a	3	7,026,091	9,513,596	Unknown		141
Foc5.1	n/a	5	26,527,434	28,923,227	Unknown		141
**Resistance to Gummy Stem Blight (GSB)**							
gsb1.1	8.7%	1	3,964,883	4,753,038	PI 183967	Mature plant resistance	105
gsb2.1	6.7%	2	28,221,008	28,901,964	PI 183967	Mature plant resistance	105
gsb3.1	6.0%	3	2,537,873	1,277,998	PI 183967	Seedling resistance	104
gsb3.2	7.4%	3	4,450,728	6,851,726	PI 183967	Seedling resistance	104
gsb4.1	6.4%	4	20,241,667	21,603,802	PI 183967	Seedling resistance	104
gsb4.1	n/a	4	22,430,156	23,641,548	HH1-8-1-2	Seedling resistance	106
gsb5.1	17.9%	5	21,932,201	23,077,375	PI 183967	Seedling resistance	104
gsb6.1	22.7%	6	3,895,743	5,175,677	PI 183967	Mature plant resistance	105
gsb6.2	8.7%	6	25,541,098	26,616,358	PI 183967	Whole stage resistance	104,105
gsb6.2	n/a	6	27,734,744	31,142,382	HH1-8-1-2	Seedling resistance	106
**Resistance to Melon Yellow Spot Virus (MYSV)**							
mysv1.1	20.1%	1	1,315,863	6,041,674	Tokiwa	*swf1.1* ; Restance to spotted wilt	107
mysv3.1	22.1%	3	5,926,467	12,326,572	Tokiwa	*swf3.1* ; Restance to spotted wilt	107
mysv4.1	13.8%	4	10,211,019	26,166,864	Tokiwa	*swf4.1* ; Restance to spotted wilt	107
mysv7.1	9.4%	7	12,693,676	1,723,853	Tokiwa	*swf7.1* ; Restance to spotted wilt	107
**Resistance to Cucurbit yellow stunting disorder virus (CYSDV)**							
cysdv5.1	n/a	5	27,072,020	32,312,548	PI 250147		108,142

* n/a: not available, or not applicable (if deteced with BSA). For early publications, the interval of a QTL was estimated from flanking markers.

Table S3. Summary of QTL for morphologic traits and abiotic stress tolerances in cucumber (as of July 2019).

Nr.	Category	Sub-category	Traits	Consensus QTL*	QTL names used in Literture	PVE (%)	Gy14 V2.0 Location Chr	Position-Left	Position-	# Populations or Environments	Reference
1	Abiotic stress tolerance	Low Temp Germination (LTG)	LTG	*LTG1.1*	*LTG1.1*	52.60%	1	18,235,527	18,827,538	065 x 02245, RILs	109
2	Abiotic stress tolerance	Low Temp Germination (LTG)	LTG	*LTG1.2*	*LTG1.2*	19.90%	1	20,227,716	22,939,356	PI197088 x Coolgreen, RILs	110
3	Abiotic stress tolerance	Low Temp Germination (LTG)	LTG	*LTG2.1*	*LTG2.1*	11.00%	2	6,206,616	8,846,704	065 x 02245, RILs	109
4	Abiotic stress tolerance	Low Temp Germination (LTG)	LTG	*LTG2.1*	*LTG2.1*	15.70%	2	6,601,244	12,748,543	PI197088 x Coolgreen, RILs	110
5	Abiotic stress tolerance	Low Temp Germination (LTG)	LTG	*LTG4.1*	*LTG4.1*	7.80%	4	1,078,420	4,109,826	065 x 02245, RILs	109
6	Abiotic stress tolerance	Low Temp Germination (LTG)	LTG	*LTG4.1*	*LTG4.1*	21.70%	4	2,363,079	4,184,374	PI197088 x Coolgreen, RILs	110
7	Abiotic stress tolerance	Waterlogging tolerance	Adventitious root number	*ARN3.1*	*ARN3.1*	3.30%	3	8,800,332	12,981,178	Zaoer-N x Pepino F2:3	111
8	Abiotic stress tolerance	Waterlogging tolerance	Adventitious root number	*ARN5.1*	*ARN5.1*	4.00%	5	20,275,230	21,836,204	Zaoer-N x Pepino F2:3	111
9	Abiotic stress tolerance	Waterlogging tolerance	Adventitious root number	*ARN6.1*	*ARN6.1*	10.40%	6	28,101,160	29,678,065	Zaoer-N x Pepino F2:3	111
10	Vegetative organ	Hypocotyl	Hypocotyl Length	*hl1.1*	*hl1.1*	10.30%	1	27,367,230	29,537,810	PI 183967 x 931 RILs	34
11	Vegetative organ	Hypocotyl	Hypocotyl Length	*hl2.1*	*hl2.1*	11.30%	2	10,842,002	16,266,343	PI 183967 x 931 RILs	34
12	Vegetative organ	Hypocotyl	Hypocotyl Length	*hl3.1*	*hl3.1*	15.10%	3	3,068,319	7,680,267	PI 183967 x 931 RILs	34
13	Vegetative organ	Hypocotyl	Hypocotyl Length	*hl3.2*	*hl3.2*	12.70%	3	27,203,767	29,471,880	PI 183967 x 931 RILs	34
14	Vegetative organ	Hypocotyl	Hypocotyl Length	*hl5.1*	*hl5.1, hl5.2, hl5.3*	13.40%	5	24,601,636	26,431,293	9110Gt x 9930, RILs	32
15	Vegetative organ	Hypocotyl	Hypocotyl Length	*hl5.1*	*hl5.1*	14.80%	5	23,964,780	24,325,543	PI 183967 x 931 RILs	34
16	Vegetative organ	Hypocotyl	Hypocotyl Length	*hl6.1*	*hl6.1*	11.40%	6	23,915,023	24,417,940	PI 183967 x 931 RILs	34
17	Vegetative organ	Hypocotyl	Hypocotyl Length	*hl6.2*	*hl6.1, hl6.2*	22.60%	6	27,227,724	31,212,546	9110Gt x 9930, RILs	32
18	Vegetative organ	Cotyledon	Cotyledon area (size)	*ca1.1*	*cl1.1, cw1.1*	12.60%	1	20,986,721	22,208,649	PI 183967 x 931 RILs	34
19	Vegetative organ	Cotyledon	Cotyledon area (size)	*ca1.2*	*cl1.1, cw1.1*	7.10%	1	27,367,230	28,141,503	9110Gt x 9930, RILs	32
20	Vegetative organ	Cotyledon	Cotyledon area (size)	*ca2.1*	*cw2.1*	8.40%	2	16,266,343	28,221,008	PI 183967 x 931 RILs	34
21	Vegetative organ	Cotyledon	Cotyledon area (size)	*ca3.1*	*cl3.1, cl3.2, cl3.3, cw.*	21.70%	3	12,985,410	18,907,727	9110Gt x 9930, RILs	32
22	Vegetative organ	Cotyledon	Cotyledon area (size)	*ca3.2*	*cl3.1*	9.70%	3	29,471,880	30,007,125	PI 183967 x 931 RILs	34
23	Vegetative organ	Cotyledon	Cotyledon area (size)	*ca5.1*	*cl5.1, cw5.1, cw5.2*	10.30%	5	17,380,728	20,738,763	9110Gt x 9930, RILs	32
24	Vegetative organ	Cotyledon	Cotyledon area (size)	*ca5.1*	*cl5.1*	23.60%	5	23,964,780	24,325,543	PI 183967 x 931 RILs	34
25	Vegetative organ	Cotyledon	Cotyledon area (size)	*ca5.1*	*cw5.1*	14.80%	5	23,077,375	24,325,543	PI 183967 x 931 RILs	34
26	Vegetative organ	Cotyledon	Cotyledon area (size)	*ca6.1*	*cl6.1*	13.30%	6	10,164,647	10,371,235	9110Gt x 9930, RILs	32
27	Vegetative organ	Cotyledon	Cotyledon area (size)	*ca6.1*	*cl6.1, cw6.1*	17.40%	6	10,767,736	24,903,405	PI 183967 x 931 RILs	34
28	Vegetative organ	Cotyledon	Cotyledon area (size)	*ca6.2*	*cw6.3*	10.30%	6	28,929,238	29,796,138	PI 183967 x 931 RILs	34
29	Vegetative organ	Leaf	Leaf area (size)	*la1.1*	*ls1.1*	n.a	1	10,000,000	18,000,000	PI 183967 x 931 RILs	33
30	Vegetative organ	Leaf	Leaf area (size)	*la2.1*	*ls2.1*	n.a	2	5,000,000	8,000,000	PI 183967 x 931 RILs	33
31	Vegetative organ	Leaf	Leaf area (size)	*la2.2*	*ls2.2*	n.a	2	27,000,000	29,000,000	PI 183967 x 931 RILs	33
32	Vegetative organ	Leaf	Leaf area (size)	*la5.1*	*fll5.1, flw5.1*	10.70%	5	21,932,201	23,964,780	PI 183967 x 931 RILs	34
33	Vegetative organ	Leaf	Leaf area (size)	*la6.1*	*flw5.1, flw6.1*	16.60%	6	28,929,238	29,796,138	PI 183967 x 931 RILs	34
34	Vegetative organ	Vine	Internode length	*il1.1*	*inl1.1*	25.00%	1	28,305,239	29,537,810	9110Gt x 9930, RILs	31
35	Vegetative organ	Vine	Internode length	*il2.1*	*inl2.1*	8.50%	2	107,777	1,718,098	9110Gt x 9930, RILs	31
36	Vegetative organ	Vine	Internode length	*il5.1*	*inl5.1*	10.00%	5	4,521,925	20,738,763	9110Gt x 9930, RILs	31
37	Vegetative organ	Vine	Internode length	*il6.1*	*inl6.1*	11.50%	6	13,360,586	22,022,192	9110Gt x 9930, RILs	31
38	Vegetative organ	Vine	Node Number (total)	*nn1.1*	*Nms 1.1*	32.70%	1	27,367,230	28,141,503	9110Gt x 9930, RILs	31
39	Vegetative organ	Vine	Vine length (plant height)	*vl1.1*	*ph1.1*	32.10%	1	27,367,230	28,141,503	9110Gt x 9930, RILs	31
40	Vegetative organ	Vine	Vine length (plant height)	*vl6.1*	*ph6.1*	9.60%	6	17,987,073	22,022,192	9110Gt x 9930, RILs	31
41	Vegetative organ	Biomass	above ground dry weight	*bio2.1*	*adw2.1*	8.80%	2	33,265,440	33,805,588	PI 183967 x 931 RILs	34
42	Vegetative organ	Biomass	above ground dry weight	*bio5.1*	*adw5.1*	8.60%	5	11,489,566	17,383,838	PI 183967 x 931 RILs	34
43	Vegetative organ	Biomass	above ground dry weight	*bio5.2*	*afw5.1*	9.60%	5	21,932,201	23,077,375	PI 183967 x 931 RILs	34
44	Vegetative organ	Biomass	above ground dry weight	*bio6.1*	*adw6.1*	8.90%	6	10,767,736	17,894,771	PI 183967 x 931 RILs	34
45	Vegetative organ	Branch	Lateral Branches Number (primary)	*lbn1.1*	*lbn1.1, flbn1.1*	27.60%	1	3,435,063	8,393,188	S94 x S06 RILs	30
46	Vegetative organ	Branch	Lateral Branches Number (primary)	*lbn1.2*	*11 qtl*	6.60%	1	28,341,342	31,105,895	S94 x S06 RILs	30
47	Vegetative organ	Branch	Lateral Branches Number (primary)	*lbn3.1*	*lbal5.1*	3.50%	3	4,419,164	5,339,800	S94 x S06 RILs	30
48	Vegetative organ	Branch	Lateral Branches Number (primary)	*lbn6.1*	*4 qtl*	8.40%	6	10,767,736	19,391,504	S94 x S06 RILs	30
49	Vegetative organ	Branch	Lateral Branches Number (primary)	*lbn6.2*	*15 qtl*	32.30%	6	22,994,885	27,270,372	S94 x S06 RILs	30
50	Vegetative organ	Branch	Lateral Branches Number (primary)	*lbn7.1*	*lbal7.1, lbtl7.1*	3.80%	7	3,274,145	11,236,180	S94 x S06 RILs	30
51	Flower	Flowering time	First flower node	*ffn1.1*	*ffn1.3 (LG)*	10.44%	1	465,777	3,986,626	S94 x S06 RILs	44
52	Flower	Flowering time	First flower node	*ffn1.2*	*ffn1.2 (LG)*	4.78%	1	16,607,265	20,239,737	S94 x S06 RILs	44
53	Flower	Flowering time	First flower node	*ffn1.3*	*ffn1.1 (LG)*	20.14%	1	28,341,342	31,105,895	S94 x S06 RILs	44
54	Flower	Flowering time	First flower node	*ffn2.1*	*ffn3.1 (LG)*	2.19%	2	27,346,384	28,646,256	S94 x S06 RILs	44
55	Flower	Flowering time	First flower node	*ffn3.1*	*ffn5.1 (LG)*	2.79%	3	0	187,602	S94 x S06 RILs	44
56	Flower	Flowering time	First flower node	*ffn3.2*	*Fpfn3.1*	18.50%	3	12,985,410	15,722,557	9110Gt x 9930, RILs	32
57	Flower	Flowering time	First flower node	*ffn5.1*	*ffn6.1 (LG)*	1.71%	5	6,670,463	7,103,094	S94 x S06 RILs	44
58	Flower	Flowering time	First flower node	*ffn5.2*	*ffn6.2 (LG)*	3.97%	5	33,369,519	33,750,000	S94 x S06 RILs	44
59	Flower	Flowering time	First flower node	*ffn6.1*	*ffn2.2 (LG)*	4.70%	6	4,310,799	2,468,105	S94 x S06 RILs	44
60	Flower	Flowering time	First flower node	*ffn6.2*	*Fpfn6.1*	37.80%	6	22,437,332	27,799,589	9110Gt x 9930, RILs	32
61	Flower	Flowering time	First flower node	*ffn6.2*	*ffn2.1*	20.97%	6	27,051,982	27,517,628	S94 x S06 RILs	44
62	Flower	Flowering time	Flowering time	*ft1.1*	*da1.1, da1.2*	16.60%	1	27,367,230	28,141,503	9110Gt x 9930, RILs	32
63	Flower	Flowering time	Flowering time	*ft1.1*	*fft1.1*	51.30%	1	26,340,943	28,305,239	CC3 x SWCC8 RILs	42
64	Flower	Flowering time	Flowering time	*ft1.1*	*fft1.1*	16.10%	1	16,279,114	32,455,127	WI7167 x WI7200 F2:3	41
65	Flower	Flowering time	Flowering time	*ft1.1*	*mft1.1*	9.10%	1	28,363,147	32,633,069	WI7167 x WI7200 F2:3	41
66	Flower	Flowering time	Flowering time	*ft5.1*	*fft5.1*	16.10%	5	28,288,488	31,530,607	WI7167 x WI7200 F2:3	41
67	Flower	Flowering time	Flowering time	*ft6.1*	*fft6.1*	61.30%	6	5,471,152	7,817,927	WI7167 x WI7200 F2:3	41
68	Flower	Flowering time	Flowering time	*ft6.2*	*fft6.2*	6.10%	6	10,371,235	11,185,947	CC3 x SWCC8 RILs	42
69	Flower	Sex expression	Multiple pistillate flowers	*mpf1.1*	*mp1.1*	8.20%	1	28,305,239	28,341,342	9110Gt x 9930, RILs	46
70	Flower	Sex expression	Multiple pistillate flowers	*mpf2.1*	*mp2.1*	13.40%	2	3,276,171	4,207,788	9110Gt x 9930, RILs	46
71	Flower	Sex expression	Multiple pistillate flowers	*mpf3.1*	*mp3.1*	11.20%	3	11,247,619	15,722,557	9110Gt x 9930, RILs	46

72	Flower	Sex expression	Multiple pistillate flowers	*mpf6.1*	*mp6.1*	13.90%	6	24,903,405	25,960,486	9110Gt x 9930, RILs	46
73	Flower	Sex expression	Multiple pistillate flowers	*mpf7.1*	*mp7.1*	11.80%	7	554,709	2,231,602	9110Gt x 9930, RILs	46
74	Flower	Sex expression	Subgynoecious	*sgy1.1*	*sg1.1*	29.00%	1	1,851,522	6,844,729	Losuas x BMB, F2/BC1	40
75	Flower	Sex expression	Subgynoecious	*sgy1.2*	*sg1.2*	18.00%	2	22,235,045	26,227,677	Losuas x BMB, F2/BC1	40
76	Flower	Sex expression	Subgynoecious	*sgy3.1*	*sg3.1*	54.60%	3	12,190,000	13,400,000	DongHuzao x S-2-98 F2/BC6	39
77	Flower	Sex expression	Subgynoecious	*sgy3.1*	*sex6.1*	5.50%	3	12315745*		H19 x G421, RILs	45
78	Flower	Sex expression	Subgynoecious	*sgy3.1*	*sg3.1*	42.00%	3	10,748,119	19,167,642	Losuas x BMB, F2/BC1	40
79	Flower	Sex expression	Subgynoecious	*sgy4.1*	*sg4.1*	n.a	4	2,900,000	6,100,000	DongHuzao x S-2-98 F2/BC6	39
80	Flower	Sex expression	Subgynoecious	*sgy5.1*	*sex6.1*	2.58%	5	32,033,873	25000000**	S94 x S06 RILs	45
81	Flower	Sex expression	Subgynoecious	*sgy6.1*	*sg6.1*	6.30%	6	9,340,981	11,112,648	DongHuzao x S-2-98 F2/BC6	39
82	Flower	Sex expression	Subgynoecious	*sgy6.1*	*sex2.2*	1.50%	6	8,837,282	10,767,736	S94 x S06 RILs	44
83	Flower	Sex expression	Subgynoecious	*sgy6.2*	*sg6.2*	4.20%	6	24,200,000	24,800,000	DongHuzao x S-2-98 F2/BC6	39
84	Flower	Sex expression	Subgynoecious	*F*	*sex1.1, sex1.2*	16.40%	6	27588957*		H19 x G421, RILs	45
85	Flower	Sex expression	Subgynoecious	*F*	*sex2.1*	60.17%	6	27,051,982	27,517,628	S94 x S06 RILs	44
86	Flower	Fruit setting	Parthenocarpic fruit set	*par1.1*	*parth1.1*	7.80%	1	26,337,208	29,537,810	EC1 x 8419s-1 F2:3	48
87	Flower	Fruit setting	Parthenocarpic fruit set	*par2.1*	*parth2.1*	5.60%	2	4,130,666	7,021,022	2A x Gy8 F2:3	47,77
88	Flower	Fruit setting	Parthenocarpic fruit set	*par2.1*	*parth2.1*	17.00%	2	3,269,283	6,309,316	EC1 x 8419s-1 F2:3	48
89	Flower	Fruit setting	Parthenocarpic fruit set	*par2.2*	*parth2.2*	7.20%	2	12,407,701	14,077,315	EC1 x 8419s-1 F2:3	48
90	Flower	Fruit setting	Parthenocarpic fruit set	*par3.1*	*parth3.1*	5.20%	3	16,948,360	21,280,153	EC1 x 8419s-1 F2:3	48
91	Flower	Fruit setting	Parthenocarpic fruit set	*par3.2*	*parth3.2*	6.40%	3	29,054,276	33,175,522	EC1 x 8419s-1 F2:3	48
92	Flower	Fruit setting	Parthenocarpic fruit set	*par4.1*	*parth4.1*	4.80%	4	27,636,080	30,643,268	2A x Gy8 F2:3	47,77
93	Flower	Fruit setting	Parthenocarpic fruit set	*par5.1*	*parth5.1*	4.10%	5	6,310,230	19,043,074	EC1 x 8419s-1 F2:3	48
94	Flower	Fruit setting	Parthenocarpic fruit set	*par5.2*	*parth5.2*	8.20%	5	24,918,882	32,540,733	2A x Gy8 F2:3	47,77
95	Flower	Fruit setting	Parthenocarpic fruit set	*par6.1*	*parth6.1*	12.20%	6	1,049,635	4,993,324	2A x Gy8 F2:3	47,77
96	Flower	Fruit setting	Parthenocarpic fruit set	*par6.2*	*parth6.2*	7.50%	6	19,624,148	22,437,332	2A x Gy8 F2:3	47,77
97	Flower	Fruit setting	Parthenocarpic fruit set	*par6.3*	*parth6.3*	13.80%	6	25,832,791	29,158,804	2A x Gy8 F2:3	47,77
98	Flower	Fruit setting	Parthenocarpic fruit set	*par7.1*	*parth7.1*	5.20%	7	1,277,240	11,866,347	2A x Gy8 F2:3	47,77
99	Flower	Fruit setting	Parthenocarpic fruit set	*par7.1*	*parth7.1*	8.90%	7	554,709	4,636,775	EC1 x 8419s-1 F2:3	48
100	Fruit	Epidermal feature	Fruit Skin wax (glaucosity)	*fsw1.1*	*wp1.1*	7.40%	1	4,604,797	6,310,491	PI 183697 x 1101, F2/BC	73
101	Fruit	Epidermal feature	Fruit Skin wax (glaucosity)	*fsw3.1*	*wp3.1*	10.30%	3	9,974,889	12,985,410	PI 183697 x 1101, F2/BC	73
102	Fruit	Epidermal feature	Fruit Skin wax (glaucosity)	*fsw5.1*	*wp5.1*	14.90%	5	25,435,033	25,749,546	PI 183697 x 1101, F2/BC	73
103	Fruit	Epidermal feature	Fruit Skin wax (glaucosity)	*fsw6.1*	*wp6.1*	18.90%	6	25,137,916	27,620,344	PI 183697 x 1101, F2/BC	73
104	Fruit	Epidermal feature	Fruit Skin wax (glaucosity)	*fsw6.2*	*wp6.2,wp6.3,wp6.4*	8.00%	6	2,674,565	5,899,013	PI 183697 x 1101, F2/BC	73
105	Fruit	Epidermal feature	Fruit spine density	*fsd4.1*	*fsd4.1*	7.80%	4	19,108,605	21,603,943	Consensus QTL from 4 populations	57
106	Fruit	Epidermal feature	Fruit spine density	*fsd6.1*	*fsd6.1*	25.70%	6	18,830,316	24,903,405	Consensus QTL from 4 populations	57
107	Fruit	Epidermal feature	Fruit spine density	*fsd6.2*	*fsd6.2*	80.60%	6	29,575,270	30,920,995	Consensus QTL from 4 populations	57
108	Fruit	Epidermal feature	Wart density	*fwd4.1*	*wd4.1*	11.00%	4	6,005,066	19,826,037	CS-PMR-1 x Santou RILs	74
109	Fruit	Epidermal feature	Wart density	*fwd5.1*	*wd5.1*	26.00%	5	26,221,218	33,529,539	CS-PMR-1 x Santou RILs	74
110	Fruit	Epidermal feature	Wart density	*fwd6.1*	*wd6.1*	9.00%	6	9,138,996	10,801,545	CS-PMR-1 x Santou RILs	74
111	Fruit	Epidermal feature	Wart Size	*fws1.1*	*ws1.1*	11.00%	1	10,041,041	18,377,790	CS-PMR-1 x Santou RILs	74
112	Fruit	Epidermal feature	Wart Size	*fws5.1*	*fws5.1, fws5.2*	68.90%	5	24,601,636	26,431,293	9110Gt x 9930, RILs	75
113	Fruit	Epidermal feature	Wart Size	*fws5.1*	*ws5.1*	44.00%	5	27,688,515	33,529,539	CS-PMR-1 x Santou RILs	74
114	Fruit	Fruit size and shape	Fruit size	*CsFS1.1*			1	9,023,276	20,536,608	Consensus QTL from 6 populations	67
115	Fruit	Fruit size and shape	Fruit size	*CsFS1.2*			1	22,623,132	30,348,448	Consensus QTL from 7 populations	67
116	Fruit	Fruit size and shape	Fruit size	*CsFS1.3*			1	3,899,897	5,366,279	Consensus QTL from 3 populations	67
117	Fruit	Fruit size and shape	Fruit size	*CsFS2.1*			2	2,481,633	15,569,877	Consensus QTL from 6 populations	67
118	Fruit	Fruit size and shape	Fruit size	*CsFS2.2*			2	31,144,770	32,619,632	Consensus QTL from 1 populations	67
119	Fruit	Fruit size and shape	Fruit size	*CsFS3.1*			3	2,439,155	8,050,295	Consensus QTL from 5 populations	67
120	Fruit	Fruit size and shape	Fruit size	*CsFS3.2*			3	28,315,821	38,377,203	Consensus QTL from 7 populations	67
121	Fruit	Fruit size and shape	Fruit size	*CsFS3.3*			3	14,413,637	23,742,682	Consensus QTL from 2 populations	67
122	Fruit	Fruit size and shape	Fruit size	*CsFS4.1*			4	19,917,090	24,886,907	Consensus QTL from 6 populations	67
123	Fruit	Fruit size and shape	Fruit size	*CsFS4.2*			4	5,003,355	17,900,808	Consensus QTL from 4 populations	67
124	Fruit	Fruit size and shape	Fruit size	*CsFS4.3*			4	26,624,946	28,337,988	Consensus QTL from 1 populations	67
125	Fruit	Fruit size and shape	Fruit size	*CsFS5.1*			5	23,964,780	26,736,192	Consensus QTL from 4 populations	67
126	Fruit	Fruit size and shape	Fruit size	*CsFS5.2*			5	28,288,488	31,530,491	Consensus QTL from 3 populations	67
127	Fruit	Fruit size and shape	Fruit size	*CsFS5.3*			5	1,274,901	4,243,996	Consensus QTL from 3 populations	67
128	Fruit	Fruit size and shape	Fruit size	*CsFS6.1*			6	20,471,682	25,710,956	Consensus QTL from 2 populations	67
129	Fruit	Fruit size and shape	Fruit size	*CsFS6.2*			6	27,620,344	31,814,492	Consensus QTL from 5 populations	67
130	Fruit	Fruit size and shape	Fruit size	*CsFS6.3*			6	9,340,981	17,987,073	Consensus QTL from 8 populations	67
131	Fruit	Fruit size and shape	Fruit size	*CsFS7.1*			7	16,055,221	22,732,146	Consensus QTL from 5 populations	67
132	Fruit	Fruit size and shape	Fruit size*	*CsFS7.2*			7	2,618,099	11,823,795	Consensus QTL from 2 populations	67
133	Fruit	Fruit size and shape	Fruit weight	*CsFW1.1*	*fw1.1*	7.50%	1	10,480,630	16,960,082	WI7167 × WI7200 $F_{2:3}$	41
134	Fruit	Fruit size and shape	Fruit weight	*CsFW1.2*	*sfw1.1*	10.60%	1	28,305,239	29,537,810	9110Gt × 9930 RILs	75
135	Fruit	Fruit size and shape	Fruit weight	*CsFW1.3*	*fw1.1 (LG)*	2.85%	1	3,435,063	3,986,626	S94 x S06 RILs	44
136	Fruit	Fruit size and shape	Fruit weight	*CsFW1.4*	*fw1.1*	20.30%	1	21,699,049	22,720,339	Gy14 × WI7221 RILs and F2:3	143
137	Fruit	Fruit size and shape	Fruit weight	*CsFW2.1*	*fw6.1 (LG)*	7.00%	2	1,747,745	2,870,181	S94 x S06 RILs	44
138	Fruit	Fruit size and shape	Fruit weight	*CsFW2.1*	*fw2.1*	5.30%	2	7,604,620	14,709,952	CC3 x SWCC8 RILs	42
139	Fruit	Fruit size and shape	Fruit weight	*CsFW3.1*	*fw5.1 (LG)*	7.05%	3	316,909	945,153	S94 x S06 RILs	44
140	Fruit	Fruit size and shape	Fruit weight	*CsFW3.2*	*fw3.1*	25.20%	3	13,514,340	15,901,514	CC3 x NC76 F2:3	70
141	Fruit	Fruit size and shape	Fruit weight	*CsFW3.3*	*fw3.2*	24.60%	3	25,262,657	30,323,040	CC3 x NC76 F2:3	70
142	Fruit	Fruit size and shape	Fruit weight	*CsFW3.3*	*fw3.1*	14.90%	3	32,595,051	35,884,128	WI7167 × WI7200 $F_{2:3}$	41
143	Fruit	Fruit size and shape	Fruit weight	*CsFW4.1*	*fw4.1 (LG)*	8.65%	4	17,993,400	19,826,037	S94 x S06 RILs	44
144	Fruit	Fruit size and shape	Fruit weight	*CsFW4.1*	*fw4.1*	7.60%	4	19,688,760	20,548,484	WI7167 × WI7200 $F_{2:3}$	41
145	Fruit	Fruit size and shape	Fruit weight	*CsFW4.2*	*fw4.1*	7.80%	4	35,493	9,004,636	Gy14 × WI7221 RILs and F2:3	143

146	Fruit	Fruit size and shape	Fruit weight	*CsFW4.2*	*fw4.1*	7.50%	4	4,989,615	9,004,636	CC3 x SWCC8 RILs	42
147	Fruit	Fruit size and shape	Fruit weight	*CsFW6.1*	*fw2.1*	11.49%	6	27,517,628	29,068,273	S94 x S06 RILs	44
148	Fruit	Fruit size and shape	Fruit weight	*CsFW6.2*	*fw6.1*	25.30%	6	10,927,954	13,883,910	CC3 x SWCC8 RILs	42
149	Fruit	Fruit size and shape	Fruit weight	*CsFW6.2*	*sfw6.1*	19.70%	6	10,893,377	17,987,073	9110Gt × 9930 RILs	75
150	Fruit	Fruit size and shape	Fruit weight	*CsFW6.2*	*fw6.1*	21.30%	6	11,677,594	20,436,346	WI7167 × WI7200 $F_{2:3}$	41
151	Fruit	Fruit size and shape	Fruit weight	*CsFW6.2*	*fw6.1*	19.10%	6	10,844,084	19,486,373	Gy14 × WI7221 RILs and F2:3	143
152	Fruit	Fruit size and shape	Fruit shape index	*CsFSI1.1*			1	8,222,187	20,446,610	Consensus QTL from 2 populations	67
153	Fruit	Fruit size and shape	Fruit shape index	*CsFSI1.2*			1	22,623,132	27,020,486	Consensus QTL from 3 populations	67
154	Fruit	Fruit size and shape	Fruit shape index	*CsFSI2.1*			2	107,777	5,183,435	Consensus QTL from 2 populations	67
155	Fruit	Fruit size and shape	Fruit shape index	*CsFSI2.2*			2	9,444,314	14,077,315	Consensus QTL from 2 populations	67
156	Fruit	Fruit size and shape	Fruit shape index	*CsFSI3.1*			3	4,093	2,809,041	Consensus QTL	67
157	Fruit	Fruit size and shape	Fruit shape index	*CsFSI3.2*			3	28,315,821	35,884,128	Consensus QTL from 2 populations	67
158	Fruit	Fruit size and shape	Fruit shape index	*CsFSI4.1*			4	6,005,066	22,207,041	Consensus QTL from 2 populations	67
159	Fruit	Fruit size and shape	Fruit shape index	*CsFSI5.1*			5	26,601,959	29,210,882	Consensus QTL	67
160	Fruit	Fruit size and shape	Fruit shape index	*CsFSI6.1*			6	4,276,910	7,817,927	Consensus QTL	67
161	Fruit	Fruit size and shape	Fruit shape index	*CsFSI6.2*			6	27,620,344	28,406,760	Consensus QTL from 2 populations	67
162	Fruit	Fruit size and shape	Fruit shape index	*CsFSI7.1*			7	14,329,677	17,191,119	Consensus QTL	67
163	Fruit	Fruit size and shape	Seed Cavity Size	*scs1.1*	*scd1.1 (LG)*	7.25%	1	3,435,063	3,986,626	S94 x S06 RILs	44
164	Fruit	Fruit size and shape	Seed Cavity Size	*scs1.2*	*scd1.2 (LG)*	4.39%	6	16,607,265	20,239,737	S94 x S06 RILs	44
165	Fruit	Fruit size and shape	Seed Cavity Size	*scs1.2*	*QTL1*	n.a	1	18,697,006	22,186,439	US2018/05490A1	144
166	Fruit	Fruit size and shape	Seed Cavity Size	*scs2.1*	*scd3.1 (LG)*	5.08%	2	1,747,745	2,870,181	S94 x S06 RILs	44
167	Fruit	Fruit size and shape	Seed Cavity Size	*scs2.1*	*QTL2*	n.a	2	346,366	4,198,858	US2018/05490A1	144
168	Fruit	Fruit size and shape	Seed Cavity Size	*scs2.2*	*scd3.2 (LG)*	4.00%	2	6,025,678	8,846,704	S94 x S06 RILs	44
169	Fruit	Fruit size and shape	Seed Cavity Size	*scs4.1*	*scd4.1 (LG)*	6.35%	4	17,993,400	19,826,037	S94 x S06 RILs	44
170	Fruit	Fruit size and shape	Seed Cavity Size	*scs5.1*	*scd6.1 (LG)*	5.60%	5	7,949,689	14,298,357	S94 x S06 RILs	44
171	Fruit	Fruit size and shape	Seed Cavity Size	*scs5.2*	*scd6.2 (LG)*	11.72%	5	16,961,924	26,736,192	S94 x S06 RILs	44
172	Fruit	Flesh	Fruit flesh thickness	*fth1.1*	*fft1.1 (LG)*	5.44%	1	3,435,063	3,986,626	S94 x S06 RILs	44
173	Fruit	Flesh	Fruit flesh thickness	*fth1.2*	*fft1.2 (LG)*	19.15%	1	16,607,265	20,239,737	S94 x S06 RILs	44
174	Fruit	Flesh	Fruit flesh thickness	*fth2.1**	*fft2.1*	42.57%	2	4,440,783	4,654,446	D8 x XUE1 F2:3	72
175	Fruit	Flesh	Fruit flesh thickness	*fth2.2*	*fft3.1 (LG)*	3.51%	2	11,896,346	14,709,952	S94 x S06 RILs	44
176	Fruit	Flesh	Fruit flesh thickness	*fth3.1*	*fft5.1 (LG)*	6.51%	3	4,419,164	5,339,800	S94 x S06 RILs	44
177	Fruit	Flesh	Fruit flesh thickness	*fth6.1*	*fft2.2, fft2.3 (LG)*	6.45%	6	7,726,325	14,870,341	S94 x S06 RILs	44
178	Fruit	Flesh	Fruit flesh thickness	*fth6.2*	*fft2.1 (LG)*	3.33%	6	27,051,982	27,270,372	S94 x S06 RILs	44
179	Fruit	Fruit size and shape	Fruit Neck Length	*fnl3.1*	*fsl5.1 (LG)*	6.42%	3	316,909	572,982	S94 x S06 RILs	44
180	Fruit	Fruit size and shape	Fruit Neck Length	*fnl3.1*	*fsl3.2*	9.70%	3	1,122,921	1,928,872	PI 183967 x 931 RILs	145
181	Fruit	Fruit size and shape	Fruit Neck Length	*fnl3.2*	*fsl3.1*	8.80%	3	13,906,742	18,907,727	PI 183967 x 931 RILs	145
182	Fruit	Fruit size and shape	Fruit Neck Length	*fnl4.1*	*fsl4.1 (LG)*	25.85%	4	18,571,199	19,826,037	S94 x S06 RILs	44
183	Fruit	Fruit size and shape	Fruit Neck Length	*fnl5.1*	*fsl6.2 (LG)*	4.24%	5	399,606	3,013,484	S94 x S06 RILs	44
184	Fruit	Fruit size and shape	Fruit Neck Length	*fnl6.1*	*fsl2.2 (LG)*	2.77%	6	13,983,867	14,800,000	S94 x S06 RILs	44
185	Fruit	Fruit size and shape	Fruit Neck Length	*fnl6.1*	*fsl6.1*	24.50%	6	11,862,392	12,289,965	9110Gt x 9930, RILs	75
186	Fruit	Fruit size and shape	Fruit Neck Length	*fnl6.2*	*fsl6.1*	24.10%	6	18,991,117	23,008,175	PI 183967 x 931 RILs	145
187	Fruit	Fruit size and shape	Fruit Neck Length	*fnl6.3*	*fsl2.1 (LG)*	5.12%	6	27,051,982	29,068,273	S94 x S06 RILs	44
188	Fruit	Fruit size and shape	Fruit Neck Length	*fnl7.1*	*fsl7.1*	11.00%	7	3,626,972	4,944,315	PI 183967 x 931 RILs	145
189	Fruit	Fruit size and shape	Fruit peduncle length	*fpl1.1*	*fpl1.1*	9.80%	1	2,706,771	4,544,991	1101 x 1694 F2	71
190	Fruit	Fruit size and shape	Fruit peduncle length	*fpl1.1*	*fpl1.1 (LG)*	4.22%	1	2,163,238	3,986,626	S94 x S06 RILs	44
191	Fruit	Fruit size and shape	Fruit peduncle length	*fpl1.2*	*fpl1.2*	9.80%	1	7,905,005	16,216,287	1101 x 1694 F2	71
192	Fruit	Fruit size and shape	Fruit peduncle length	*fpl3.1*	*fpl5.1 (LG)*	2.86%	3	14,258,579	16,797,391	S94 x S06 RILs	44
193	Fruit	Fruit size and shape	Fruit peduncle length	*fpl3.2*	*fpl5.2 (LG)*	5.87%	3	29,502,335	32,624,601	S94 x S06 RILs	44
194	Fruit	Fruit size and shape	Fruit peduncle length	*fpl5.1*	*fpl6.1 (LG)*	8.87%	5	25,378,536	32,033,873	S94 x S06 RILs	44
195	Fruit	Fruit size and shape	Fruit peduncle length	*fpl6.1*	*fpl2.1 (LG)*	2.66%	6	13,983,867	14,653,826	S94 x S06 RILs	44
196	Fruit	Fruit size and shape	Fruit peduncle length	*fpl6.2*	*fpl6.1*	21.50%	6	25,960,456	27,143,384	1101 x 1694 F2	71
197	Fruit	Fruit size and shape	Fruit peduncle length	*fpl6.2*	*fpl2.2 (LG)*	6.79%	6	27,270,372	27,517,628	S94 x S06 RILs	44
198	Seed	Seed	Seed Size	*sds2.1*	*sl2.1, swd2.1, 100sw*	18.80%	2	30,222,371	32,798,977	PI 183967 x 931 RILs	76
199	Seed	Seed	Seed Size	*sds3.1*	*sl3.1*	7.70%	3	35,165,748	38,000,000	PI 183967 x 931 RILs	76
200	Seed	Seed	Seed Size	*sds3.1*	*sds-3*	12.25%	3	33,290,019	40,652,814	2A x Gy8 F2:3	77
201	Seed	Seed	Seed Size	*sds4.1*	*sl4.1*	9.20%	4	9,092,431	17,900,808	PI 183967 x 931 RILs	76
202	Seed	Seed	Seed Size	*sds4.1*	*sds-4, 50swt-4*	11.87%	4	2,530,050	11,841,341	2A x Gy8 F2:3	77
203	Seed	Seed	Seed Size	*sds5.1*	*sl5.1*	15.60%	5	4,068,484	24,325,543	PI 183967 x 931 RILs	76
204	Seed	Seed	Seed Size	*sds5.1*	*swd5.1*	16.20%	5	9,966,733	12,185,473	PI 183967 x 931 RILs	76
205	Seed	Seed	Seed Size	*sds5.1*	*100swt5.1*	13.40%	5	3,608,355	26,736,192	PI 183967 x 931 RILs	76
206	Seed	Seed	Seed Size	*sds5.1*	*sds-5, 50swt-5*	10.09%	5	11,526,950	24,325,543	2A x Gy8 F2:3	77
207	Seed	Seed	Seed Size	*sds6.1*	*sl6.1, 100swt6.1*	9.10%	6	16,419,103	18,934,337	PI 183967 x 931 RILs	76
208	Seed	Seed	Seed Size	*sds6.1*	*swd6.1*	9.90%	6	13,373,768	16,419,103	PI 183967 x 931 RILs	76
209	Seed	Seed	Seed Size	*sds6.2*	*sds-6, 50swt-6*	5.32%	6	23,629,402	32,122,847	2A x Gy8 F2:3	77
210	Seed	Seed	Seed Size	*sds7.1*	50swt-7	8.83%	7	4,636,775	11,866,347	2A x Gy8 F2:3	77
211	MISC	Regeneration ability	Regeneration ability on MS medium	*ra1.1*	*fcrms1.1*	n.a	1	5,658,180	6,299,507	9110Gt x 9930, RILs	146
212	MISC	Regeneration ability	Regeneration ability on MS medium	*ra3.1*	*fcrms3.1*	n.a	3	30,104,525	30,484,658	9110Gt x 9930, RILs	146
213	MISC	Regeneration ability	Regeneration ability on MS medium	*ra6.1*	*fcrms6.1*	n.a	6	4,264,786	5,612,251	9110Gt x 9930, RILs	146

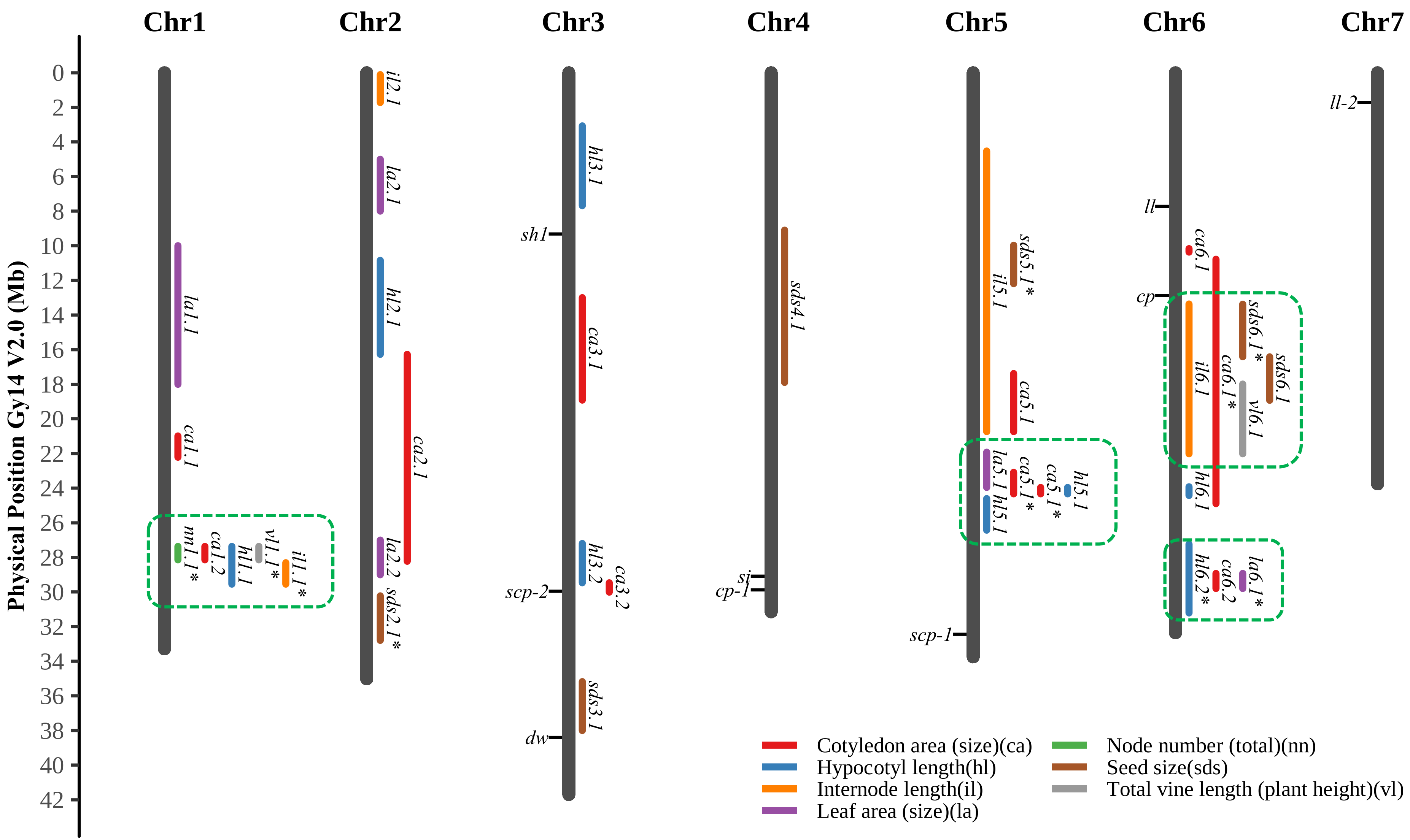

Supplementary File 4 (Figure S1). Chromosomal locations of QTL for vegetative growth- and development-related traits in cucumber. Ruler to the left indicates locations (in Mbp) in the Gy14 V2.0 draft genome assembly (drawn to scale). Vertical black lines are chromosomes. Cloned genes and QTL are aligned to the left and right of each chromosome, respectively. Vertical bar for each QTL represents 1.5 or 2.0 LOD confidence interval on the chromosome. Rectangles indicate clusters of QTL.

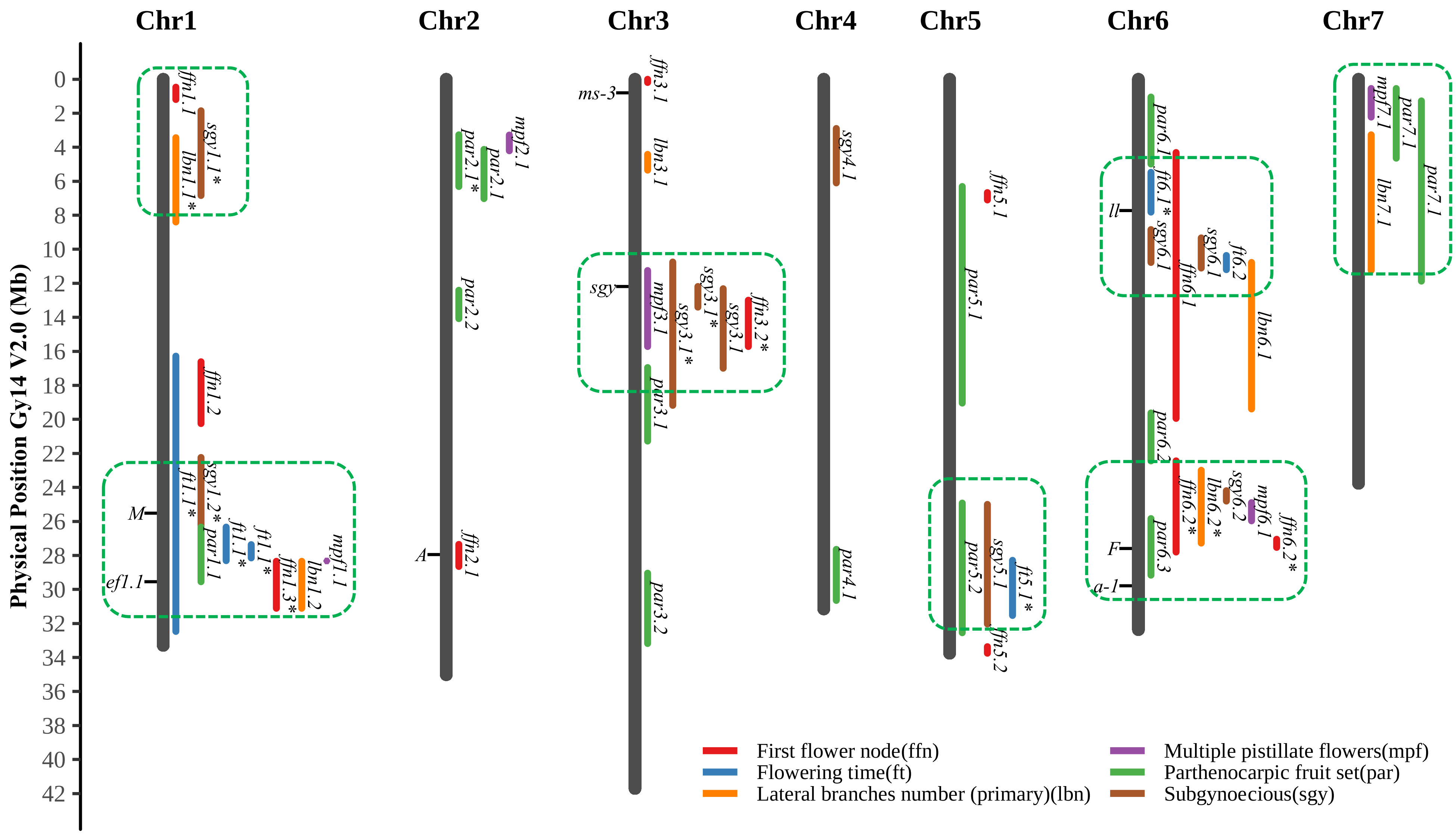

Supplementary File 5 (Figure S2). Chromosomal locations of QTL for flower-related traits (sex expression and fruit set) in cucumber. Ruler to the left indicates locations (in Mbp) in the Gy14 V2.0 draft genome assembly (drawn to scale). Vertical black lines are chromosomes. Cloned genes and QTL are aligned to the left and right of each chromosome, respectively. Vertical bar for each QTL represents 1.5 or 2.0 LOD confidence interval on the chromosome. Rectangles indicate clusters of QTL.

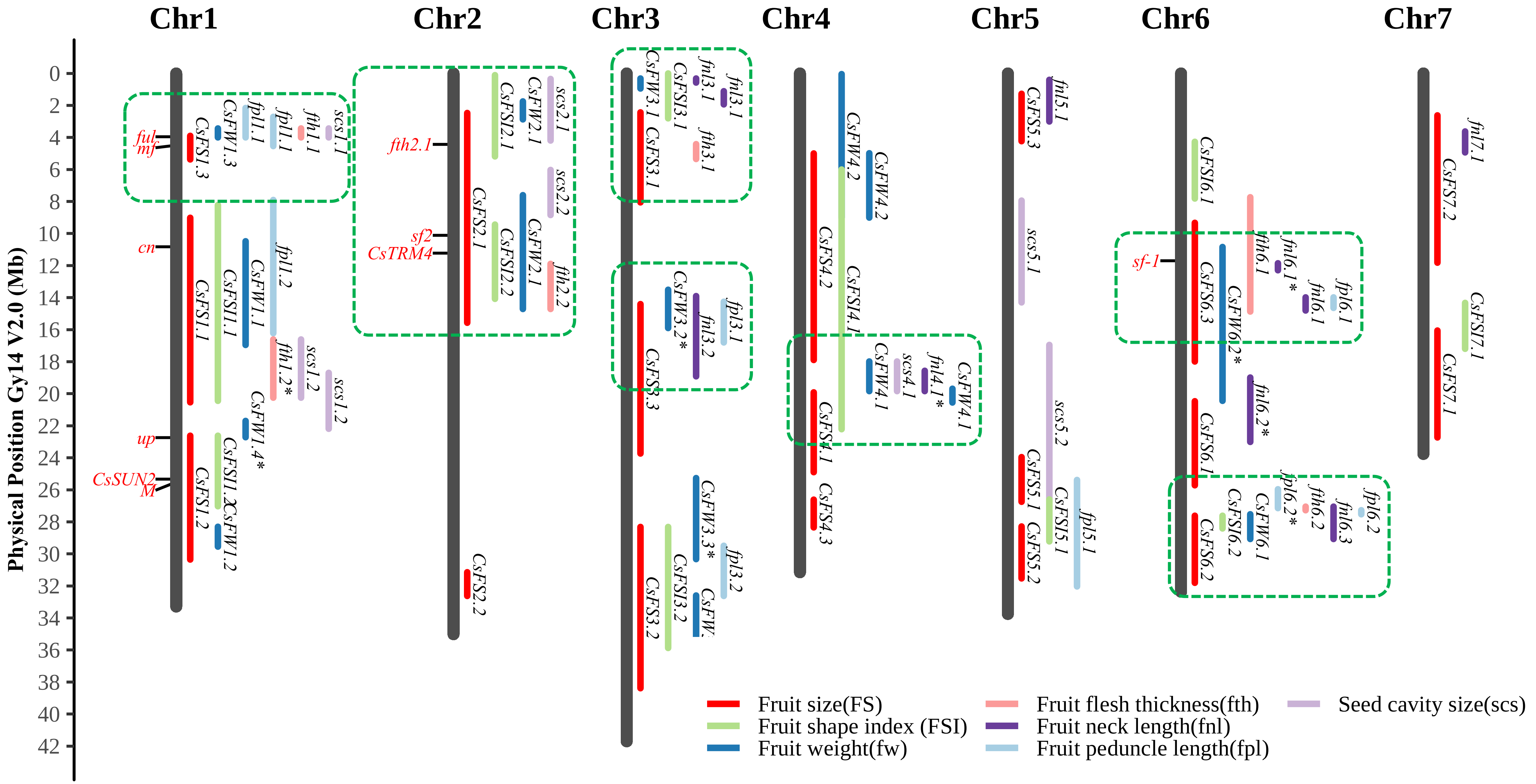

Supplementary File 6 (Figure S3). Chromosomal locations of fruit-related QTL in cucumber. Ruler to the left indicates locations (in Mbp) in the Gy14 V2.0 draft genome assembly (drawn to scale). Vertical black lines are chromosomes. Cloned genes and QTL are aligned to the left and right of each chromosome, respectively. Vertical bar for each QTL represents 1.5 or 2.0 LOD confidence interval on the chromosome. Rectangles indicate clusters of QTL.

Supplementary File 7. Additional references cited in supplementary files (continued from text):

115. Shi, T., Wang, S., Lin, T., Yang, Q. & Huang, S. Genetic mapping of little leaf 2 (*ll2*), a major QTL controlling leaf area in cucumber (*Cucumis sativus* L.). *J. Agric. Biotechnol.* **22**, 415–421 (2014).
116. Calderon, C. I., Yandell, B. S. & Havey, M. J. Genetic mapping of paternal sorting of mitochondria in cucumber. *Theor. Appl. Genet.* **125**, 11–8 (2012).
117. Gao, D. *et al.* Mutation in a novel gene *SMALL AND CORDATE LEAF 1* affects leaf morphology in cucumber. *J. Integr. Plant Biol.* **59**, 736–741 (2017).
118. Yong, J. *et al.* The simple sequence repeat (SSR) and sequence-tagged sites (STS) markers linked to the compact gene (*cp*) in cucumber (Cucumis sativus L.). *J. Agric. Biotechnol.* **21**, 1152–1158 (2013).
119. Han, Y. *et al.* Fine mapping of a male sterility gene *ms-3* in a novel cucumber (*Cucumis sativus* L.) mutant. *Theor. Appl. Genet.* **131**, 449–460 (2018).
120. Boualem, A. *et al.* A conserved ethylene biosynthesis enzyme leads to andromonoecy in two Cucumis species. *PLoS One* **4**, e6144 (2009).
121. Sun, J. *et al.* Mapping and identification of *CsUp*, a gene encoding an Auxilin-like protein, as a putative candidate gene for the *upward-pedicel* mutation (*up*) in cucumber. *BMC Plant Biol.* **19**, 1–12 (2019).
122. Zhou, Q. *et al.* A sequencing-based linkage map of cucumber. *Mol. Plant* **8**, 961–963 (2015).
123. Miao, H. *et al.* A linkage map of cultivated cucumber (*Cucumis sativus* L.) with 248 microsatellite marker loci and seven genes for horticulturally important traits. *Euphytica* **182**, 167–176 (2011).
124. Zhang, S. *et al.* Molecular mapping and candidate gene analysis for fruit epidermal structure in cucumber. *Plant Breed.* **136**, 767–774 (2017).
125. Yang, X. *et al.* Fine mapping of the uniform immature fruit color gene u in cucumber (*Cucumis sativus* L.). *Euphytica* **196**, 341–348 (2014).
126. Liu, H. *et al.* Fine genetic mapping of the white immature fruit color gene *w* to a 33.0-kb region in cucumber (*Cucumis sativus* L.). *Theor. Appl. Genet.* **128**, 2375–2385 (2015).
127. Hao, N. *et al. CsMYB36* is involved in the formation of yellow green peel in cucumber (*Cucumis sativus* L.). *Theor. Appl. Genet.* **131**, 1659–1669 (2018).
128. Yang, X. *et al.* High-resolution mapping of the dull fruit skin gene *D* in cucumber (*Cucumis sativus* L.). *Mol. Breed.* **33**, 15–22 (2014).
129. Liu, P. *et al.* Molecular mapping and candidate gene analysis for resistance to powdery mildew in Cucumis sativus stem. *Genet. Mol. Res.* **5**, gmrl16039680 (2017).
130. Yang, S. Genetic analysis and mapping of *cca-2* gene resistant to cucumber target leaf spot. (Chinese Academy of Agricultural Sciences, 2012).
131. Pramnoi, P., Somta, P., Chankaew, S., Juwattanasomran, R. & Srinives, P. A single recessive gene controls fragrance in cucumber (*Cucumis sativus* L .). *Indian Acad. Sci.* **92**, 147–149 (2013).
132. Zhang, P., Zhu, Y., Wang, L., Chen, L. & Zhou, S. Mining candidate genes associated with powdery mildew resistance in cucumber via super-BSA by specific length amplified fragment (SLAF) sequencing. *BMC Genomics* **16**, 1–14 (2015).
133. Zhang, H. *et al.* SSR markers linked to the resistant gene of cucumber powdery mildew.

Acta Agric. Boreali-Sinica **23**, 77–80 (2008).
134. Bai, Z. *et al.* QTL mapping of resistance gene to downy mildew in cucumber. *Prog Nat Sci* **18**, 706–710 (2008).
135. Zhang, S. *et al.* Chromosomal mapping and QTL analysis of resistance to downy mildew in Cucumis sativus. *Plant Dis.* **97**, 245–251 (2013).
136. Caldwell, D. *et al.* Methods and compositions for identifying downy mildew resistant cucumber plants. (2011).
137. Win, K. T., Vegas, J., Zhang, C., Song, K. & Lee, S. QTL mapping for downy mildew resistance in cucumber via bulked segregant analysis using next-generation sequencing and conventional methods. *Theor. Appl. Genet.* **130**, 199–211 (2017).
138. Szczechura, W., Staniaszek, M., Klosinska, U. & Kozik, E. U. Molecular analysis of new sources of resistance to *Pseudoperonospora cubensis* (Berk. et Curt.) Rostovzev in cucumber. *Russ. J. Genet.* **51**, 974–979 (2015).
139. Pang, X., Zhou, X., Wan, H. & Chen, J. QTL mapping of downy mildew resistance in an introgression line derived from interspecific hybridization between cucumber and *Cucumis hystrix*. *J. Phytopathol.* **161**, 536–543 (2013).
140. Słomnicka, R. *et al.* Genetic mapping of *psl* locus and quantitative trait loci for angular leaf spot resistance in cucumber (*Cucumis sativus* L.). *Mol. Breed.* **38**, (2018).
141. Haaring, C., Spyropoulos, A. & Lastdrader, M. B. QTLs for fusarium resistance in cucumber. (2018).
142. de Ruiter, W., Hofstede, R., de Vries, J. & van den Heuvel, H. Combining QTLs for resistance to CYSDV and powdery mildew in a single cucumber line. *Cucurbitaceae 2008, Proc. IXth EUCARPIA Meet. Genet. Breed. Cucurbitaceae* 181–188 (2008).
143. Sheng, Y., Pan, Y., Li, Y., Yang, L. & Weng, Y. Quantitative trait loci for fruit size and flowering time-related traits under domestication and diversifying selection in cucumber (Cucumis sativus L.). *Plant Breed.* (**submitted**).
144. Haaring, C. & Huijbregts-doorduin, L. J. Genetic basis for cucumber fruit having small seed cavity. (2018).
145. Wang, M. *et al.* Quantitative trait loci associated with fruit length and stalk length in cucumber using RIL population. *Acta Bot Boreal-Occident Sin* **34**, 1764–1770 (2014).
146. Wang, Y. *et al.* Genetic analysis and identification of a candidate gene associated with in vitro regeneration ability of cucumber. *Theor. Appl. Genet.* **131**, 2663–2675 (2018).
